# Genetic sequencing for surveillance of drug resistance in tuberculosis in highly endemic countries: a multi-country population-based surveillance study

**DOI:** 10.1016/S1473-3099(18)30073-2

**Published:** 2018-06

**Authors:** Matteo Zignol, Andrea Maurizio Cabibbe, Anna S Dean, Philippe Glaziou, Natavan Alikhanova, Cecilia Ama, Sönke Andres, Anna Barbova, Angeli Borbe-Reyes, Daniel P Chin, Daniela Maria Cirillo, Charlotte Colvin, Andrei Dadu, Andries Dreyer, Michèle Driesen, Christopher Gilpin, Rumina Hasan, Zahra Hasan, Sven Hoffner, Alamdar Hussain, Nazir Ismail, S M Mostofa Kamal, Faisal Masood Khanzada, Michael Kimerling, Thomas Andreas Kohl, Mikael Mansjö, Paolo Miotto, Ya Diul Mukadi, Lindiwe Mvusi, Stefan Niemann, Shaheed V Omar, Leen Rigouts, Marco Schito, Ivita Sela, Mehriban Seyfaddinova, Girts Skenders, Alena Skrahina, Sabira Tahseen, William A Wells, Alexander Zhurilo, Karin Weyer, Katherine Floyd, Mario C Raviglione

**Affiliations:** a Global Tuberculosis Programme, World Health Organization, Geneva, Switzerland; b San Raffaele Scientific Institute, Milan, Italy; c Scientific Research Institute of Lung Diseases, Ministry of Health, Baku, Azerbaijan; d National Tuberculosis Reference Laboratory, Manila, Philippines; e National Reference Laboratory for Mycobacteria, Borstel Research Centre, Borstel, Germany; f Molecular and Experimental Mycobacteriology, Borstel Research Centre, Borstel, Germany; g Central Reference Laboratory on Tuberculosis Microbiological Diagnostics, Ministry of Health, Kiev, Ukraine; h Bill & Melinda Gates Foundation, Seattle, WA, USA; i Bureau for Global Health, US Agency for International Development, Washington, DC, USA; j Regional Office for Europe, World Health Organization, Copenhagen, Denmark; k National Institute for Communicable Diseases, Sandringham, South Africa; l Mycobacteriology Unit, Institute of Tropical Medicine, Antwerp, Belgium; m Department of Pathology and Laboratory Medicine, Aga Khan University, Karachi, Pakistan; n Department of Microbiology, Tumour and Cell Biology, Karolinska Institute, Stockholm, Sweden; o National Reference Laboratory, National Tuberculosis Control Programme, Islamabad, Pakistan; p Department of Medical Microbiology, University of Pretoria, Pretoria, South Africa; q Department of Pathology and Microbiology, National Institute of Diseases of the Chest and Hospital, Dhaka, Bangladesh; r KNCV Tuberculosis Foundation, The Hague, Netherlands; s Department of Microbiology, Public Health Agency of Sweden, Solna, Sweden; t Tuberculosis Control and Management Unit, National Department of Health, Pretoria, South Africa; u Department of Biomedical Sciences, University of Antwerp, Antwerp, Belgium; v Critical Path Institute, Tucson, AZ, USA; w Department of Mycobacteriology, Tuberculosis and Lung Disease Centre, Riga East University Hospital, Riga, Latvia; x Republican Scientific and Practical Centre for Pulmonology and Tuberculosis, Minsk, Belarus; y National Institute of Phthisiology And Pulmonology, National Academy of Medical Science of Ukraine, Kiev, Ukraine

## Abstract

**Background:**

In many countries, regular monitoring of the emergence of resistance to anti-tuberculosis drugs is hampered by the limitations of phenotypic testing for drug susceptibility. We therefore evaluated the use of genetic sequencing for surveillance of drug resistance in tuberculosis.

**Methods:**

Population-level surveys were done in hospitals and clinics in seven countries (Azerbaijan, Bangladesh, Belarus, Pakistan, Philippines, South Africa, and Ukraine) to evaluate the use of genetic sequencing to estimate the resistance of *Mycobacterium tuberculosis* isolates to rifampicin, isoniazid, ofloxacin, moxifloxacin, pyrazinamide, kanamycin, amikacin, and capreomycin. For each drug, we assessed the accuracy of genetic sequencing by a comparison of the adjusted prevalence of resistance, measured by genetic sequencing, with the true prevalence of resistance, determined by phenotypic testing.

**Findings:**

Isolates were taken from 7094 patients with tuberculosis who were enrolled in the study between November, 2009, and May, 2014. In all tuberculosis cases, the overall pooled sensitivity values for predicting resistance by genetic sequencing were 91% (95% CI 87–94) for *rpoB* (rifampicin resistance), 86% (74–93) for *katG, inhA*, and *fabG* promoter combined (isoniazid resistance), 54% (39–68) for *pncA* (pyrazinamide resistance), 85% (77–91) for *gyrA* and *gyrB* combined (ofloxacin resistance), and 88% (81–92) for *gyrA* and *gyrB* combined (moxifloxacin resistance). For nearly all drugs and in most settings, there was a large overlap in the estimated prevalence of drug resistance by genetic sequencing and the estimated prevalence by phenotypic testing.

**Interpretation:**

Genetic sequencing can be a valuable tool for surveillance of drug resistance, providing new opportunities to monitor drug resistance in tuberculosis in resource-poor countries. Before its widespread adoption for surveillance purposes, there is a need to standardise DNA extraction methods, recording and reporting nomenclature, and data interpretation.

**Funding:**

Bill & Melinda Gates Foundation, United States Agency for International Development, Global Alliance for Tuberculosis Drug Development.

## Introduction

Antimicrobial resistance represents a threat to global health and security and challenges the progress made over the past two decades to combat tuberculosis.^1^ In 2016, WHO estimated that there were 10·4 million incident cases of tuberculosis.^2^ Furthermore, about 600 000 cases of multidrug-resistant tuberculosis or of rifampicin-resistant disease were estimated to have emerged in the same year.^2^ Surveillance of drug resistance, which has both public and individual health consequences, is recognised to be a crucial component of tuberculosis control programmes worldwide. Surveillance data can be used to estimate disease burden, plan diagnostic and treatment services, monitor the effectiveness of tuberculosis control interventions, design effective standard treatment regimens and, if systematically undertaken, allow individually tailored patient care.

Global surveillance of resistance to anti-tuberculosis drugs, which was launched in 1994, is the oldest and largest global surveillance project of antimicrobial resistance.^3^ In most countries with a high burden of tuberculosis, there is inadequate capacity for routine testing of all patients for resistance to drugs. In these settings, the extent of drug resistance is estimated through periodic epidemiological surveys, which have largely relied on conventional culture and drug susceptibility testing by use of phenotypic methods. These surveys have important limitations, including the need for timely refrigerated transportation to preserve the bacterial viability of samples and minimise contamination, suboptimal reproducibility of tests, a long turnaround for results, a requirement for effective biosafety precautions in the operating laboratories, and high workloads for the reference laboratories. These factors make it difficult to repeat surveys at regular intervals.^3^

**Research in context**

**Evidence before this study**

Surveillance of resistance to anti-tuberculosis drugs is needed to estimate disease burden, plan diagnostic and treatment services, monitor the effectiveness of tuberculosis control interventions, design effective standard treatment regimens and, ultimately, allow the most appropriate, individually tailored patient care. We searched MEDLINE for articles published between Jan 1, 1966, and Sept 20, 2017, and Embase for articles published between Jan 1, 1980, and Sept 20, 2017. To find articles about the use of genetic sequencing for surveillance of drug resistance in tuberculosis, we used the search terms “tuberculosis”, “drug resistance”, “surveillance”, and “genome sequencing” to find publications in English, French, or Spanish. In most countries with a high burden of tuberculosis, there is insufficient capacity to routinely test all patients for resistance to anti-tuberculosis drugs. In these settings, the burden of drug-resistant tuberculosis is estimated through periodic epidemiological surveys. For more than 20 years, these surveys have relied on conventional culture and drug susceptibility testing by use of phenotypic methods. These methods have crucial limitations, including the need to preserve bacterial viability of samples, suboptimal reproducibility of tests, discordant testing results between assays, and the requirement for effective biosafety. All these factors make it difficult to repeat surveys at regular intervals, particularly in resource-poor countries. Genome sequencing is a high-throughput technology that is mainly used in research settings and for surveillance purposes in high-income countries. To the best of our knowledge, genome sequencing has never been evaluated as a method for surveillance of drug resistance in tuberculosis in resource-poor countries.

**Added value of this study**

Our Article presents the results of the first ever population-based surveys to investigate the use of genome sequencing for surveillance of the resistance of *Mycobacterium tuberculosis* to key anti-tuberculosis drugs (rifampicin, isoniazid, ofloxacin, moxifloxacin, pyrazinamide, kanamycin, amikacin, and capreomycin) in resource-limited countries. Although most other genome sequencing studies focus on patient groups that are at risk of drug resistance, we present data from patients with different risks of drug resistance, including patients who have recently been diagnosed and those who have already been treated for tuberculosis across different epidemiological settings. Our work offers insights into the feasibility of introducing high-throughput genome sequencing technology to replace conventional phenotypic testing for surveillance of drug resistance in tuberculosis.

**Implications of the available evidence**

Our results have implications for the future surveillance of drug resistance in tuberculosis and encourage the use of genome sequencing for broader surveillance of antimicrobial resistance. For drugs with suboptimal sensitivity of genome sequencing compared with phenotypic testing in the general patient population, the true prevalence of drug resistance can be determined using a relatively simple statistical adjustment. Our Article shows that genome sequencing is a valuable tool for surveillance of drug resistance in resource-poor settings and could potentially replace phenotypic testing in drug resistance surveys. Ultimately, these findings could allow the establishment of a comprehensive continuous surveillance system for drug resistance, even in settings with limited laboratory capacity. The findings of this study can also be used to guide the development and introduction of new diagnostic technologies in different geographical areas and patient groups and contribute to overall knowledge of the role of genotypic markers in conferring resistance to anti-tuberculosis drugs.

Implementation of routine diagnostic testing for drug susceptibility, which is recommended by WHO as part of their End TB Strategy, remains insufficient. In 2016, global surveillance data showed that only 33% of new patients and 61% of patients who were previously treated for tuberculosis had access to drug susceptibility testing at the time of diagnosis.^2^ Although the rollout of rapid molecular diagnostics, particularly GeneXpert MTB/RIF (Cepheid, Sunnyvale, CA, USA), is starting to increase access to susceptibility testing for rifampicin resistance, much greater efforts are needed to meet the WHO target of universal drug susceptibility testing.^4^

Considering the slow growth rate of *Mycobacterium tuberculosis* complex strains, molecular-based testing could be the only way to obtain rapid test results regarding drug susceptibility for many patients. There is therefore an urgent need for rapid and easy-to-perform molecular tests of drug susceptibility for a wide range of anti-tuberculosis drugs.^5^ Additionally, the distribution of such tests requires a clear set of criteria to interpret molecular test results, which should be based on an understanding of the association between genotypic markers of resistance, phenotypic test results, and patient outcomes.

Previous studies^6^, ^7^ have summarised current knowledge about the correlation between genotypic markers and phenotypic test results. An important limitation of current evidence is that data are generally restricted to patients with a high risk of drug resistance who have already received treatment for tuberculosis. There is little evidence for a correlation between genotypic markers and phenotypic test results in the context of population-based surveillance, particularly among newly diagnosed patients with no known pre-existing risk of drug resistance. Such data are crucial for predicting the accuracy of new molecular tests to accurately diagnose drug resistance in patient groups with different prevalence of resistance, different pre-existing risks (such as in patients who have been treated several times), or both.^5^ Additionally, data on the use of genetic sequencing for surveillance of anti-tuberculosis drug resistance and tuberculosis outbreak investigations are primarily from industrialised countries.^8^ Data from resource-limited settings remain scarce.

We present results from a unique population-based surveillance study across seven resource-limited countries with a high burden of tuberculosis. The efficacy of genetic sequencing to estimate the extent of resistance of *M tuberculosis* isolates to the major first-line and second-line anti-tuberculosis drugs (namely, rifampicin, isoniazid, ofloxacin, moxifloxacin, pyrazinamide, kanamycin, amikacin, and capreomycin) was investigated.

## Methods

### Study design and participants

Between November, 2009, and May, 2014, population-based surveys to measure anti-tuberculosis drug resistance of *M tuberculosis* isolates were done in hospitals and clinics in Azerbaijan,^9^ Bangladesh,^10^ Belarus (Minsk),^11^ Pakistan,^12^ Philippines,^13^ South Africa (Gauteng and Kwazulu Natal provinces),^14^ and Ukraine.^15^ Details on the design of these surveys are provided elsewhere.^16^ All patients with pulmonary tuberculosis, both newly diagnosed and previously treated, who presented to the study sites were eligible for enrolment. Site selection was done by cluster sampling or by inclusion of all diagnostic facilities in the country. The results of each study are described in the individual country reports.^9^, ^10^, ^11^, ^12^, ^13^, ^14^, ^15^, ^17^ Each study obtained ethical approval and written informed consent from patients.

### Procedures

Strains of *M tuberculosis* were isolated from sputum samples in either Löwenstein-Jensen media or a BACTEC MGIT 960 liquid culture system (Becton Dickinson, Franklin Lakes, NJ, USA). The laboratory methods used in each country and additional details about methods are provided in the appendix. Methods were standardised and all laboratories successfully passed proficiency testing before survey initiation. The number of isolates that underwent phenotypic susceptibility testing varied between drugs, as reported in the results section. Phenotypic susceptibility testing involved growing mycobacteria in the presence of antibiotics; if a colony formed, the mycobacteria were deemed to be resistant.

Genetic sequencing data were obtained either through whole-genome sequencing or targeted gene sequencing of the relevant genomic regions of *rpoB* for rifampicin, *katG, inhA*, and *fabG* promoter for isoniazid, *pncA* for pyrazinamide, and *gyrA* and *gyrB* for the fluoroquinolones^18^ (ofloxacin and moxifloxacin) by use of Sanger technology (Thermo Fisher Scientific, Waltham, MA, USA). Multi-drug-resistant tuberculosis was defined as resistance to at least rifampicin and isoniazid, and extensively drug-resistant tuberculosis was defined as multidrug-resistant tuberculosis with additional resistance to at least one fluoroquinolone and one second-line injectable drug (kanamycin, amikacin, or capreomycin). For whole-genome sequencing of isolates, either Illumina technology (Illumina, San Diego, CA, USA) or Ion Torrent technology (Thermo Fisher Scientific) were used. The appendix presents the sequencing methods used in each country. Details on DNA extraction, sequencing methods, and primers used for Sanger sequencing are provided in the appendix. Whole-genome sequencing data were submitted to the Sequence Read Archive of the National Center for Biotechnology Information as recalibrated BAM files (accession number SRP128089). The interpretation of mutations in *rpoB, katG, inhA, fabG* promoter, *pncA, gyrA, gyrB, rrs,* and *ei*s genes was done with a standardised approach for grading mutations in *M tuberculosis* in terms of their association with drug resistance.^6^ By this approach, the assessor classified the level of confidence that a given mutation was associated with resistance as either “high confidence for association with resistance”, “moderate confidence for association with resistance”, “minimal confidence for association with resistance”, “no association with resistance”, or “indeterminate” (appendix).

The average cost of doing genetic sequencing in the study was calculated and compared with the average cost of first-line and second-line phenotypic drug susceptibility testing.

### Statistical analysis

The accuracy of genetic sequencing compared with phenotypic test results was assessed for the following genes: *rpoB* for rifampicin; *katG, inhA*, and *fabG* promoter for isoniazid; *pncA* for pyrazinamide; *gyrA* and *gyrB* for ofloxacin and moxifloxacin; *rrs* and *eis* for kanamycin; and *rrs* for amikacin and capreomycin. Pooled distributions for the sensitivity and specificity of the tests based on the genotypic method were obtained using random effects modelling after logistic transformation and use of a restricted maximum likelihood estimator.

Mutations classified in the high confidence, moderate confidence, or minimal confidence categories^6^ were considered to be conferring true resistance, even if the phenotypic testing showed susceptibility. For these cases, it was assumed that any phenotypic test results that indicated susceptibility were false negatives. The specificity of sequencing was thus set at 100%. Throughout this Article, when reference is made to the true prevalence of resistance as determined using phenotypic testing, this adjustment is accounted for.

Where θ denotes the apparent prevalence of drug resistance, measured by use of genomic sequencing, and φ denotes the bias-corrected (true) prevalence, determined by use of phenotypic testing, φ is expressed in terms of θ, sensitivity (*se*), and specificity (*sp*), as the formula:

$$\varphi =\frac{\theta +sp-1}{se+sp-1}$$

Uncertainty about *se* and *sp* and uncertainty around θ (due to sampling) were propagated. Uncertainty was propagated using a Bayesian model, which was implemented in JAGS 4.3.0.^19^ To set up the model, it was assumed that *se* and *sp* followed a beta distribution. Parameters were obtained by use of the method of moments.^20^ An uninformative prior was set at φ. The likelihood function was obtained by solving this equation for θ, using *sp* set to 1, and pooled *sp* values separately in the equation:

L(φ, se, sp|θ)=seφ

The conditional probability distribution of φ was proportional to the product of the likelihood and the prior,

Prob(φ|θ) a L(φ|θ)Prob(φ)

from which summary statistics were extracted.

### Role of the funding source

DPC is an employee of the Bill & Melinda Gates Foundation, which co-funded the study. CC, YDM, and WAW are employees of the United States Agency for International Development, which also co-funded the study. These authors were acting as subject matter experts rather than agency representatives, and did not have veto power over any study decision. The corresponding author had full access to all the data in the study and had final responsibility for the decision to submit for publication.

## Results

Of 7094 patients enrolled in the study, 751 (11%) were from Azerbaijan, 949 (13%) from Bangladesh, 197 (3%) from Belarus, 1461 (21%) from Pakistan, 1017 (14%) from the Philippines, 1578 (22%) from South Africa, and 1141 (16%) from Ukraine. Among all patients enrolled, 5611 (79%) were new tuberculosis patients, 1278 (18%) were previously treated, and the remaining 205 (3%) had an unknown treatment history for tuberculosis. Of the 7094 patients, 6124 (86%) had rifampicin-susceptible tuberculosis and 970 (14%) had rifampicin-resistant tuberculosis. The number of patients with available data for both genotypic and phenotypic testing of *M tuberculosis* complex isolates varied between drugs, from 7010 for rifampicin to 623 for kanamycin (table). Data on resistance to injectable drugs (kanamycin, amikacin, and capreomycin), and on pyrazinamide resistance in Ukraine, were only available for patients with rifampicin-resistant tuberculosis. Data on the quality of sequencing results are reported in the appendix. The overall pooled sensitivity values for genetic sequencing among all tuberculosis cases were 91% (95% CI 87–94) for *rpoB* (rifampicin resistance), 86% (74–93) for *katG, inhA*, and *fabG* promoter combined (isoniazid resistance), 85% (77–91) for *gyrA* and *gyrB* combined (ofloxacin resistance), and 88% (81–92) for *gyrA* and *gyrB* combined (moxifloxacin resistance). The sensitivity for *pncA* (pyrazinamide) compared with MGIT 960 testing, adjusted for the results of the Wayne's test, was 54% (39–68). Sensitivity for multidrug-resistant tuberculosis (resistance to rifampicin and isoniazid) was 85% (75–91), and sensitivity for extensively drug-resistant tuberculosis (multidrug-resistant tuberculosis and resistance to at least one fluoroquinolone and one second-line injectable drug) was 74% (53–87).

**Table:** Number of clinical *Mycobacterium tuberculosis* isolates tested and the pooled sensitivity values of genetic sequencing compared with phenotypic testing, stratified by rifampicin resistance status, for each locus or the loci conferring resistance to the indicated drug

	**Loci**	**Rifampicin-susceptible cases**		**Rifampicin-resistant cases**		**All cases**	
		Number of isolates	Sensitivity (95% CI)	Number of isolates	Sensitivity (95% CI)	Number of isolates	Sensitivity (95% CI)
Rifampicin	*rpoB*	..	..	..	..	7010	91% (87–94)
Isoniazid	*katG, inhA*, and *fabG* promoter	6065	81% (66–90)	953	90% (81–95)	7018	86% (74–93)
Ofloxacin	*gyrA* and *gyrB*	4244	76% (51–90)	866	88% (83–92)	5110	85% (77–91)
Moxifloxacin	*gyrA* and *gyrB*	4010	81% (53–94)	783	91% (85–95)	4793	88% (81–92)
Pyrazinamide	*pncA*	2310	37% (22–54)	683	55% (40–70)	2993	51% (35–66)
Pyrazinamide*	*pncA*	2310	50% (33–67)	683	54% (40–68)	2993	54% (39–68)
Kanamycin	*rrs* and *eis*	..	..	623	79% (58–91)	..	..
Amikacin	*rrs*	..	..	690	90% (82–95)	..	..
Capreomycin	rrs	..	..	764	81% (56–93)	..	..
Multidrug-resistant	NA	..	..	..	..	6986	85% (75–91)
Extensively drug-resistant	NA	..	..	..	..	756	74% (53–87)

* Adjusted with Wayne's test results.

Sensitivity values were always higher for rifampicin-resistant than rifampicin-susceptible isolates, but values were highly variable by setting and patient group (appendix). Variations in the sensitivity of genetic sequencing across geographical sites were greatest among rifampicin-susceptible cases for all drugs and in pyrazinamide-resistant isolates in all patient groups.

A detailed description of the mutations that were observed to be associated with resistance of each drug is reported in the appendix. False-negative phenotypic test results (ie, the isolates carrying mutations considered to define true resistance even in the presence of a result of phenotypic susceptibility) occurred in 87 (9%) of 958 rifampicin-resistant strains, 57 (4%) of 1519 isoniazid-resistant strains, 12 (3%) of 353 ofloxacin-resistant strains, 59 (19%) of 318 moxifloxacin-resistant strains, and nine (2%) of 479 pyrazinamide-resistant strains of all tuberculosis cases. Among patients with rifampicin-resistant tuberculosis, false-negative phenotypic test results occurred in 16 (10%) of 163 kanamycin-resistant strains, five (4%) of 124 amikacin-resistant strains, and 23 (17%) of 136 capreomycin-resistant strains.

**Figure 1:**
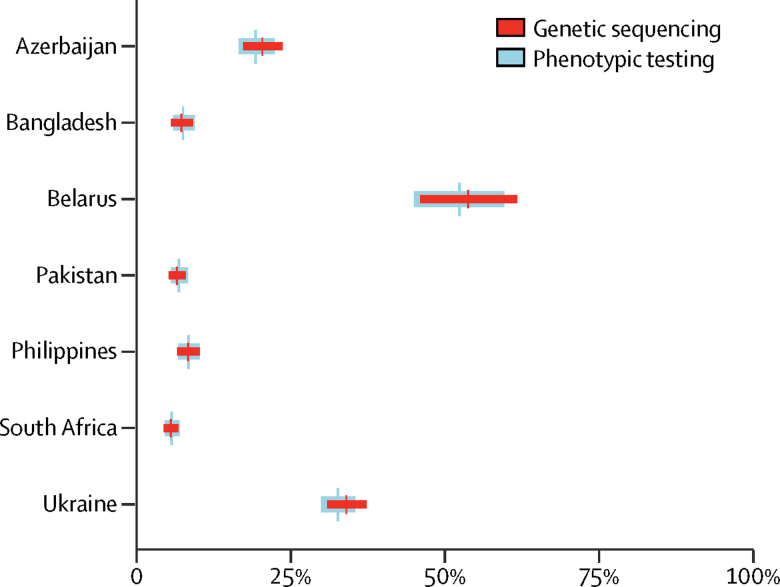
Prevalence of rifampicin resistance, estimated through genetic sequencing compared with phenotypic testing Data are the adjusted prevalence of rifampicin resistance from genetic sequencing compared with the true prevalence of rifampicin resistance from phenotypic testing, shown for all tuberculosis cases. Absolute numbers are shown in the appendix.

**Figure 2:**
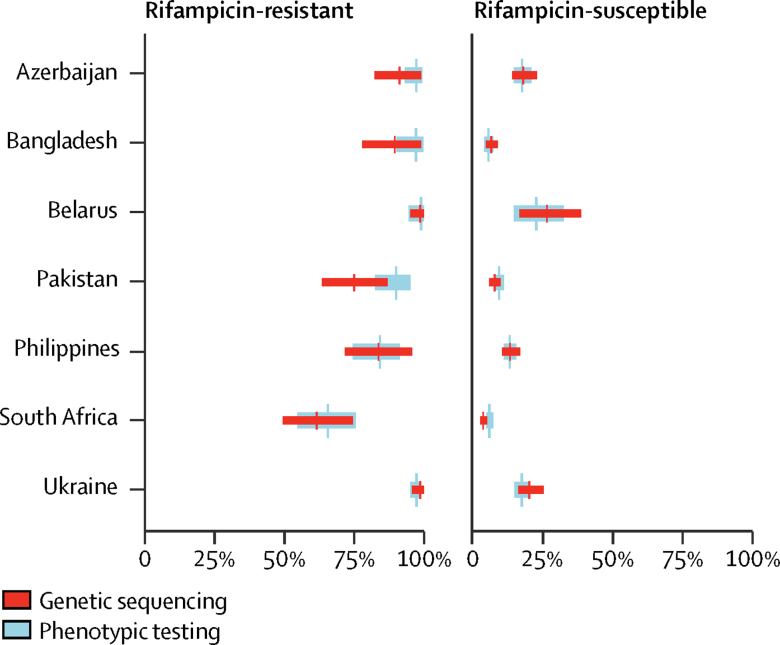
Prevalence of isoniazid resistance, estimated through genetic sequencing compared with phenotypic testing Data are the adjusted prevalence of isoniazid resistance from genetic sequencing compared with the true prevalence of isoniazid resistance from phenotypic testing, stratified by rifampicin-resistant and rifampicin-susceptible cases. Absolute numbers are shown in the appendix.

**Figure 3:**
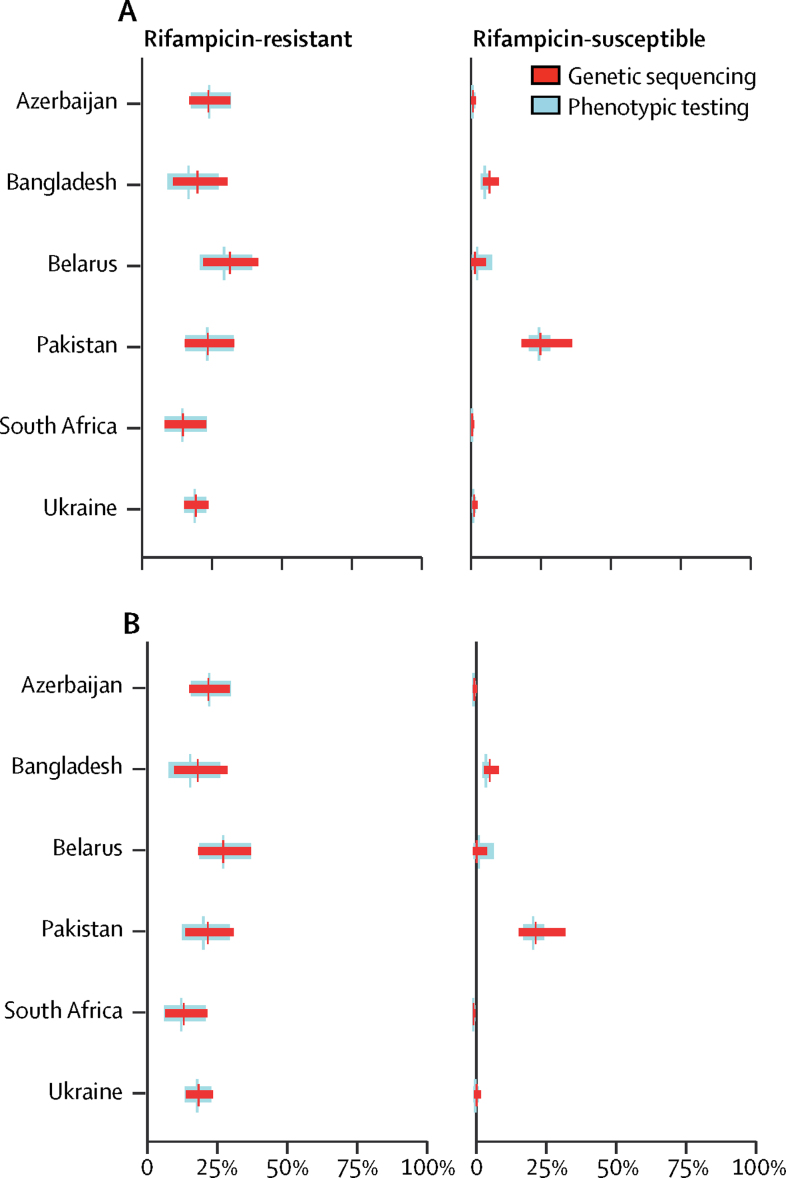
Prevalence of fluoroquinolone resistance, estimated through genetic sequencing compared with phenotypic testing Data are the adjusted prevalence of fluoroquinolone resistance from genetic sequencing compared with the true prevalence of fluoroquinolone resistance from phenotypic testing, stratified by (A) ofloxacin and (B) moxifloxacin resistance and rifampicin-resistant and rifampicin-susceptible cases. Absolute numbers are shown in the appendix.

**Figure 4:**
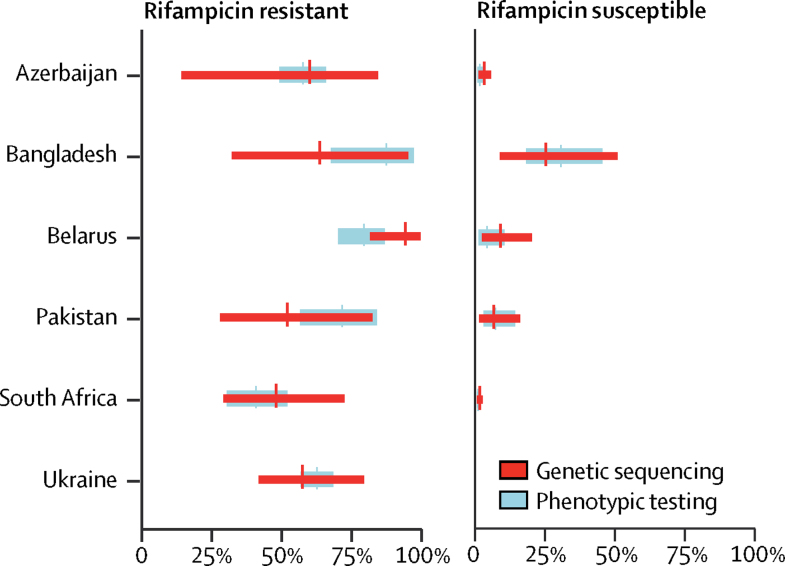
Prevalence of pyrazinamide resistance, estimated through genetic sequencing compared with phenotypic testing Data are the adjusted prevalence of pyrazinamide resistance from genetic sequencing compared with the true prevalence of pyrazinamide resistance from phenotypic testing, stratified by rifampicin-resistant and rifampicin-susceptible cases. Absolute numbers are shown in the appendix.

**Figure 5:**
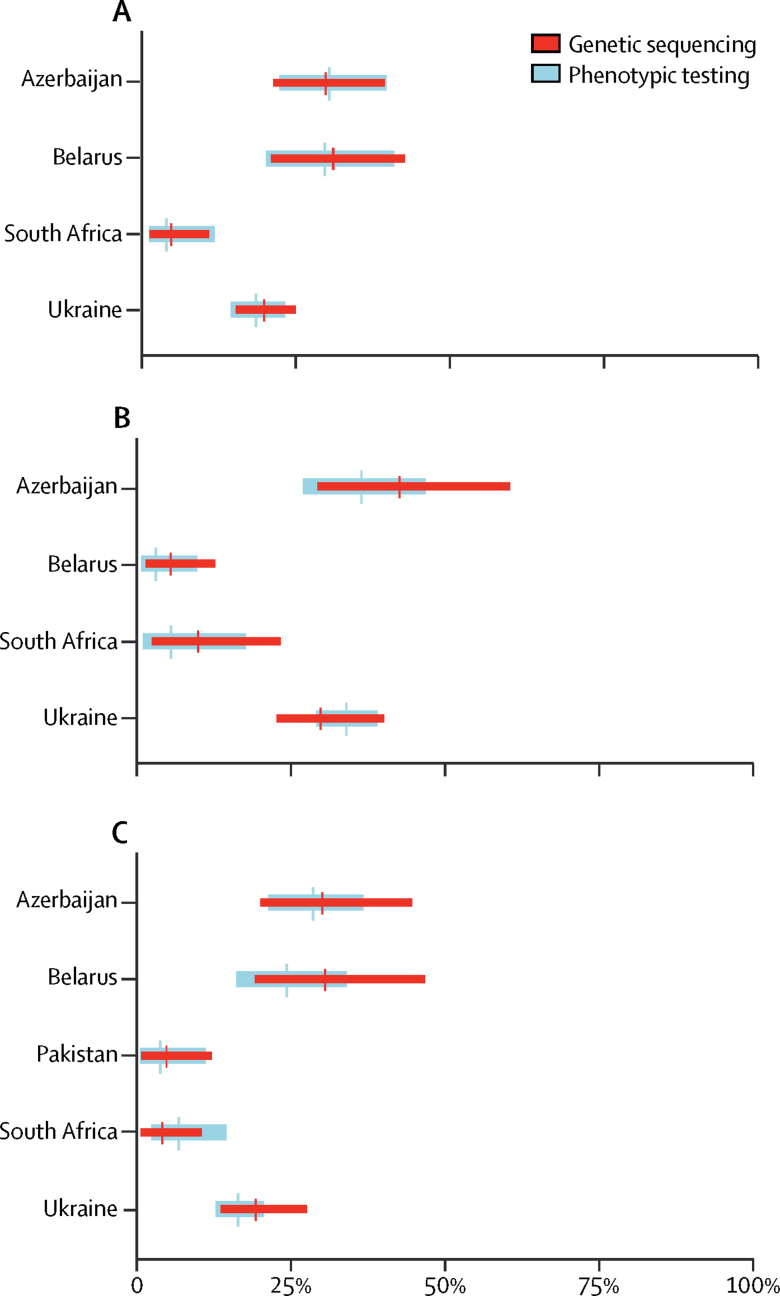
Prevalence of resistance to injectable kanamycin, amikacin, and capreomycin, estimated through genetic sequencing compared with phenotypic testing Data are the adjusted prevalence of resistance to injectable drugs, determined from genetic sequencing, compared with the true prevalence of resistance to injectable drugs from phenotypic testing, stratified into (A) kanamycin, (B) amikacin, and (C) capreomycin resistance, and are shown in rifampicin-resistant cases. Absolute numbers are shown in the appendix.

Comparisons between the prevalence of drug resistance, estimated with genetic sequencing after adjustment for the sensitivity of sequencing, and the true prevalence of drug resistance, determined with phenotypic testing, were made for each drug (Figure 1, Figure 2, Figure 3, Figure 4, Figure 5; appendix). There was a large overlap between resistance determined by genetic sequencing and the true prevalence of drug resistance. We stratified results into rifampicin-resistant and rifampicin-susceptible tuberculosis for most drugs except for the injectable drugs, because data regarding resistance to these drugs were only available for rifampicin-resistant cases.

The average cost of genome sequencing was US$150 per sample. Drug susceptibility testing was done in two rounds, first with first-line drugs and then with second-line drugs. The average cost of conventional drug susceptibility testing to rifampicin, isoniazid, ofloxacin, moxifloxacin, pyrazinamide, kanamycin, amikacin, and capreomycin was $230. These costs included the cost of kits, reagents, and staff time.

## Discussion

We have presented the results of a surveillance project of more than 7000 patients across several countries to assess the accuracy of genetic sequencing in determining the prevalence of resistance to the most commonly used first-line and second-line anti-tuberculosis drugs when compared with phenotypic testing. We found that genetic sequencing can be a valuable surveillance tool to accurately predict drug resistance in low-income and middle-income countries. The value of this work is that the isolates tested are representative of the entire tuberculosis patient population in seven resource-limited countries, all of which are classified as having a high burden of tuberculosis or multidrug-resistant tuberculosis. The settings also differed in their risk of and extent of drug resistance. These patients were managed under several programmatic and epidemiological situations, and included patients who had been newly diagnosed with tuberculosis and patients who had previously been treated for tuberculosis.

Our results show that the accuracy of genetic sequencing is very good at predicting phenotypic resistance to rifampicin, isoniazid, the fluoroquinolones, and (among rifampicin-resistant cases) injectable drugs. These findings imply that the sensitivity values of sequencing compared with phenotypic testing (table) can be applied to sequencing results to estimate the true prevalence of drug resistance for surveillance purposes. These sensitivity values are consistent with previously published evidence.^6^, ^7^

One of the most difficult aspects of any study on accuracy of a diagnostic technology is defining the gold-standard test to be used as comparator. Phenotypic tests are typically used as comparator to access the accuracy of genetic tests. In assessing the drug resistance of tuberculosis, the reliability and reproducibility of phenotypic tests are suboptimal for some drugs, and clinical decisions are often made by use of a combination of phenotypic and genotypic test results.^21^, ^22^, ^23^ In our Article, we considered the phenotypic test to be the gold-standard test; however, in the event of phenotypic test results finding drug susceptibly alongside the presence of mutations considered to be markers of resistance, the genetic test result was assumed to be correct. Given the nature of this large surveillance project, the discrepancies between phenotypic and sequencing results could not be investigated by repeating the phenotypic test. For all discrepancies, the sequencing data were thoroughly reviewed. For pyrazinamide, all strains with discrepant results were reassessed by use of a third method (Wayne's test) on the basis of the poor reproducibility of phenotypic tests.

The breadth of sensitivity values observed across sites (appendix) reflects differences in the quality of phenotypic testing, random fluctuations due to test errors (particularly when the number of resistant cases was very small), and variation in the prevalence of resistance to rifampicin. Some of these differences could be partly explained by the *M tuberculosis* genetic background, correlation with specific drug resistance mutations, and clonal spread within geographical areas.

Variations in the sensitivity of sequencing, which were most pronounced for pyrazinamide and among rifampicin-susceptible cases, resulted in large uncertainty bounds around the estimates of prevalence by sequencing. To accurately monitor trends in drug resistance, more work should be done to improve the sensitivity of sequencing, particularly among rifampicin-susceptible cases.

A larger difference between the prevalence of resistance as estimated by phenotypic testing and the adjusted prevalence by sequencing occurred when the sensitivity of genotypic testing was either notably higher or lower than in the other countries. For example, in Pakistan, the sensitivity of sequencing for isoniazid was lower than in other countries (figure 2), whereas the sensitivity of genotypic testing for pyrazinamide in Belarus (figure 4) was higher than elsewhere.

Pyrazinamide is the drug for which the ability of genetic sequencing to predict phenotypic resistance was most problematic, as shown in figure 4 by the poor overlap of prevalence estimates generated by phenotypic testing and by sequencing, particularly among rifampicin-susceptible strains from Belarus and Pakistan, and by large uncertainty bounds around the sequencing-based prevalence estimates. This finding was unsurprising because our understanding of the role of mutations in conferring resistance to pyrazinamide is incomplete: 42% of all *pncA* mutations were unclassified in our dataset. Although most of these mutations have already been reported in the literature to be associated with resistance, their infrequent occurrence means that there is insufficient statistical power to classify them.^6^, ^22^ Additionally, the phenotypic test for pyrazinamide has inadequate reproducibility, making it a weak test with which to make comparisons.^22^, ^23^

The frequency of mutations and associated phenotypic drug susceptibility results from our study can be used for genome-based predictions of resistance (appendix). To further improve our understanding of genotypic markers of resistance, phenotypic and genotypic data should be considered in the context of clinical outcome data, given the suboptimal reliability and reproducibility of phenotypic tests for some anti-tuberculosis drugs and uncertainty around the most appropriate critical concentrations.^24^ To accelerate the transition from reliance on phenotypic results to genotypic results for resistance prediction, it is crucial that genotypic, phenotypic, and outcome data be shared as soon as they become available.

The main purpose of surveillance is to estimate the burden of drug resistance and monitor its trends, to enable a prompt and effective public health response. Our findings show that genotypic methods have an important role in surveillance, especially given the limitations of conventional phenotypic methods, and that available molecular diagnostic tools can only detect resistance to a small number of drugs. Targeted gene sequencing or whole-genome sequencing directly on sputum samples would bypass the need for culture, standardise the approach, and accelerate the availability of results.^25^ When genetic sequencing is properly standardised and made economically feasible, this technology could be particularly impactful in countries with low laboratory and sample referral capacity, and would offer an opportunity to monitor the development of drug resistance more effectively during tuberculosis epidemics. Enabling use of gene sequencing would also represent a breakthrough in improving rapid surveillance of both drug-resistant tuberculosis and broader antimicrobial resistance.

The cost of genetic sequencing will be a major factor in determining the feasibility of introduction and the speed of expansion. Costs of sequencing are progressively decreasing and are already lower than the cost of phenotypic testing to first-line and second-line anti-tuberculosis drugs in most settings.^26^ This trend has also been confirmed in our study. In the context of surveillance, the possibility of grouping specimens to genome sequence many isolates (up to 200) in one single run offers the potential for further cost savings and a reduction in laboratory workload.

Besides cost, there are several other technical challenges that need to be addressed to allow widespread use of genetic sequencing for surveillance of drug resistance in tuberculosis. DNA extraction and sample preparation methods need to be consistent, standardised nomenclature to record and report sequencing information must be developed, an external quality assurance system for genome sequencing (similar to the system available for phenotypic testing) should be established, and a standardised and common approach to analysis must be defined.^3^, ^27^, ^28^, ^29^ To support the expansion of the use of sequencing technologies in low-income and middle-income countries, molecular biology and bioinformatics skills should be developed locally and supplemented with continuous mentoring from supranational reference laboratories.

Although constraints for the expansion of genetic sequencing exist, the value of whole genome sequencing data is enormous for any national tuberculosis control programme. Data can be re-analysed at a later stage to investigate newly discovered resistance-associated loci, to predict resistance to new drugs, and to inform development of more tailored molecular diagnostics as soon as knowledge on the genetic marker of resistance becomes available.

Our study has some limitations. First, excepting isolates with graded mutations, which were all assumed to be phenotypically resistant as previously described,^6^ we considered phenotypic drug susceptibility testing to be the gold-standard test, and we treated this test as a comparator for genetic sequencing. However, there is evidence that phenotypic drug susceptibility testing might not always be the most accurate test. Treatment outcome data should be used to guide interpretation of genotypic and phenotypic testing results^21^ and, unfortunately, these data were not available in our study. Second, phenotypic testing was done at specific critical concentrations, as currently recommended by WHO,^30^ but more recent evidence suggests that some of these concentrations might need to be reassessed.^24^ Third, although only laboratory methods recommended by WHO were used and all laboratories passed proficiency testing before beginning the project, some variability in phenotypic results between laboratories and between media types could have affected outcomes. Fourth, different platforms were used for DNA sequencing, including Sanger platforms, which could have slightly different coverage of some genomic regions.^31^ Finally, given that testing for second-line injectable drugs was limited to strains with rifampicin resistance, the number of isolates tested for kanamycin, amikacin, and capreomycin is not large enough to generate conclusive evidence.

Our work shows that genetic sequencing can be a valuable tool in surveillance of drug resistance, enabling new ways to monitor drug resistance in tuberculosis in low-income and middle-income countries. The findings of this study can also be used to guide the development and introduction of new diagnostic technologies, including genetic sequencing, in different geographical areas and patient groups and contribute to our knowledge on the role of genotypic markers in conferring resistance to anti-tuberculosis drugs.

**This online publication has been corrected. The corrected version first appeared at thelancet.com/infection on March 27, 2018**
