# Corrections

**DOI:** 10.1016/S1473-3099(18)30227-5

**Published:** 2018-05

*Zignol M, Maurizio Cabibbe A, Dean AS, et al. Genetic sequencing for surveillance of drug resistance in tuberculosis in highly endemic countries: a multi-country population-based surveillance study.* Lancet Infect Dis *2018; published online March 21. https://doi.org/10.1016/S1473-3099(18)30073-2.* The key for figure 4 was incorrect, and should have read “Genetic sequencing” and “Phenotypic testing”. This correction has been made to the online version as of March 27, 2018, and the printed Article will be corrected.

