# Supplementary material for: Genetic sequencing for surveillance of drug resistance in tuberculosis in highly endemic countries: a multi-country population-based surveillance study
Source: Lancet Infect Dis. 2018 Jun;18(6):675–83. doi: 10.1016/S1473-3099(18)30073-2 (PMC5968368; doi:10.1016/S1473-3099(18)30073-2)
Supplement: Supplementary appendix [file mmc1.pdf]

THE LANCET Infectious Diseases

Supplementary webappendix

This webappendix formed part of the original submission and has been peer reviewed. We post it as supplied by the authors.

Supplement to: Zignol M,Cabibbe AM, Dean AS, et al. Genetic sequencing for surveillance of drug resistance in tuberculosis in highly endemic countries: a multi-country population-based surveillance study. *Lancet Infect Dis* 2018; published online March 21. http://dx.doi.org/10.1016/S1473-3099(18)30073-2.

Appendix: Genetic sequencing for surveillance of drug resistance in tuberculosis in highly endemic countries: a multi-country population-based study

Methods

Phenotypic testing

Identification of *M. tuberculosis* complex was performed using biochemical and immunochromatographic tests [1]. Susceptibility testing was conducted using the Löwenstein-Jensen (LJ) proportion method or BACTEC MGIT 960 (Becton Dickinson, Sparks, MD, USA) with 1% as the critical proportion (for details by country see supplement Table 1, Appendix, page 7) according to manufacturers' instructions, at the following critical concentrations recommended by the World Health Organization for LJ and MGIT 960, respectively [2]: rifampicin, 40.0 µg/mL and 1.0 µg/mL; isoniazid, 0.2 µg/mL and 0.1 µg/mL; pyrazinamide, 100.0 µg/mL (MGIT 960); ofloxacin, 4.0 µg/mL and 2.0 µg/mL; moxifloxacin, 0.5 µg/mL (MGIT 960); kanamycin, 30.0 µg/mL and 2.5 µg/mL; amikacin, 30.0 µg/mL and 1.0 µg/mL; capreomycin, 40.0 µg/mL and 2.5 µg/mL. Strains showing discrepant results between *pncA* sequencing and phenotypic testing of pyrazinamide in MGIT 960 were additionally tested using the Wayne enzymatic pyrazinamidase assay. The pyrazinamidase assay was performed as described by Wayne, with slight modifications as previously published [3].

DNA extraction

To extract and purify high-quality genomic DNA for next generation sequencing, commercially available (para)magnetic- (Maxwell 16 Cell DNA Purification kit, Promega, Madison, WI, USA; NucliSENS easyMag, Biomerieux, Marcy l'Etoile, France), and column-based (QIAamp DNA Mini Kit, Qiagen, Hilden, Germany) systems, or CTAB (N-cetyl-N,N,N-trimethyl ammonium bromide)/NaCl protocol were used according to manufacturer's instructions and published procedures [4],[5]. When Sanger sequencing was used, DNA was extracted from pure cultures by thermal lysis and sonication as described elsewhere [6].

DNA sequencing

Three different technologies were used for DNA sequencing.

1. Illumina technology (Illumina Inc., San Diego, CA, USA). Paired-end libraries were prepared using the Nextera XT DNA Sample Preparation kit and sequenced on the Hiseq 2500, NextSeq 500 and MiSeq platforms (reads length 101 bp, 151 bp and 251 bp, respectively), according to the manufacturer's instructions.
2. Ion PGM technology (Thermo Fisher Scientific Inc., Waltham, MA, USA). Library and emulsion PCR were performed following manufacturer's instructions (reads length: 400 bp).

The reads generated with these two next generation sequencing technologies were checked for quality and aligned to *M. tuberculosis* H37Rv reference genome (GenBank AL123456.3). In particular, total variant calling was performed by using the CLC Genomics Workbench 7 tool (Qiagen, Hilden, Germany) for Ion PGM technology with the following filters: minimum reads coverage: 10, minimum count of reads calling variants: 2, minimum frequency of reads calling variants: 10%, minimum frequency of reads calling variants in each direction: 5%. Pyro-error variants in homopolymer regions with a minimum length of 3 and a frequency below 0.8 were removed. A published on-line analysis pipeline was applied to ensure quality of variant calls for sequences generated by Illumina technology [7] with the following checks: no unusual depth (>2× compared to average coverage), no additional variant calls within 12 base pairs of another SNP or indel, at least 5 reads calling variants, consensus on >75% reads including at least one read for each direction. In addition, a standardized analysis pipeline developed by the laboratories participating in the study was used to confirm and cross-check the results. A package of bioinformatics software was used for recalibration, indels realignment and variant calling with the following filters: 4 reads covering each direction, a total of 4 reads with a phred score greater or equal to 20 and allele frequency greater or equal to 75%. Moreover, in the event of doubtful calls, reads mapping to the reference genome were visually inspected. When analysis of at least the targeted genomic regions was not possible due to low

quality or insufficient reads coverage of sequencing results, the test was repeated or the sample excluded.

3. Sanger technology (Thermo Fisher Scientific Inc.) The relevant genomic regions of *rpoB*, *katG*, *fabG* promoter, *inhA*, *pncA*, *gyrA* and *gyrB* were targeted [8]. The primers used are reported in Supplement Table 2. PCR products were sequenced using 3730xl platform (Thermo Fisher Scientific Inc.), according to manufacturer's instructions.

The output results were assembled and analysed using ClustalW application (BioEdit software, Ibis Biosciences, Abbott Company, Carlsbad, CA, USA) and Sequencher software (Sequencher version 4.9 DNA sequence analysis software, Gene Codes Corporation, Ann Arbor, MI USA). Sequences were aligned to the *M. tuberculosis* H37Rv reference strain (GenBank AL123456.3) to identify nucleotide mutations. Chromatograms with poor quality were re-sequenced and were re-analyzed if the reads improved. Any discordant results were re-analyzed.

Nucleotide variants detected by the different sequencing technologies were annotated based on the *M. tuberculosis* H37Rv reference genome. Mutations in *gyrB* gene were reported according to the annotation proposed by Camus et al, locating the Quinolone Resistance-Determining Region (QRDR) between codons 461 and 499 [9].

The role of variants detected within the targeted genomic regions was assigned according to the recently developed, first-ever standardized approach for the grading of mutations in *M. tuberculosis* according to their association with drug resistance [10]. Specifically, a standardised procedure for grading drug resistance-associated mutations based on positive likelihood ratios (LR) and thresholds commonly adopted in evidence-based medicine was used for objectively evaluating whether mutations were positively or negatively associated with phenotypic resistance. Using this rationale, mutations were graded as having either "high" ($p < 0.05$, $LR > 10$), "moderate" ($p < 0.05$, $5 < LR \leq 10$) or "minimal" confidence ($p < 0.05$, $1 < LR \leq 5$) of being associated with resistance, or as "not associated with resistance" ($p < 0.05$, $LR < 1$) or as "indeterminate" ($p \geq 0.05$). In addition, systematic reviews and available databases were used to infer the role of mutations not classified with the grading system

and for substitutions not linked to resistance [7,8,11,12,13,14,15].

In accordance with the abovementioned approach described by Miotto *et al*. [10], mutations classified as having high, moderate, and minimal confidence were assumed as true markers or resistance, overruling the phenotypic result whenever such mutations were observed.

Results

Quality of sequencing results

A reads coverage relative to the reference genome of 69.2x was obtained on average from samples undergoing whole genome sequencing, with a mean percentage of unambiguous coverage relative to the complete reference genome of 96%. Specifically, more than 95% of whole genome sequenced samples achieved a reads coverage of at least 20x, as minimum threshold accepted to analyse results with high accuracy (and 86% of these achieved more than 30x). Considering the two different next generation sequencing technologies used, a 30x mean coverage was achieved, at least, from both.

References

1 Kent P, Kubica G. Public health mycobacteriology: a guide for the level III laboratory. U.S Dept of Health and Human Services. Public Health Service, Centers for Disease Control, Atlanta, GA, USA. 1985.

2 Updated interim critical concentrations for first-line and second-line DST. Geneva, World Health Organization, 2012.

3 Singh P, Wesley C, Jadaun GPS, *et al.* Comparative evaluation of Löwenstein-Jensen proportion method, BacT/ALERT 3D system, and enzymatic pyrazinamidase assay for pyrazinamide susceptibility testing of Mycobacterium tuberculosis. *J Clin Microbiol* 2007; **45**: 76–80.

4 Käser M, Ruf M-T, Hauser J, Marsollier L, Pluschke G. Optimized method for preparation of DNA from pathogenic and environmental mycobacteria. *Appl Environ Microbiol* 2009; **75**: 414–8.

5 Köser CU, Bryant JM, Becq J, *et al.* Whole-genome sequencing for rapid susceptibility testing of M. tuberculosis. *N Engl J Med* 2013; **369**: 290–2.

6 Zwadyk P, Down JA, Myers N, Dey MS. Rendering of mycobacteria safe for molecular diagnostic studies and development of a lysis method for strand displacement amplification and PCR. *J Clin Microbiol* 1994; **32**: 2140–6.

7 Feuerriegel S, Schleusener V, Beckert P, *et al.* PhyResSE: a Web Tool Delineating Mycobacterium tuberculosis Antibiotic Resistance and Lineage from Whole-Genome Sequencing Data. *J Clin Microbiol* 2015; **53**: 1908–14.

8 Zhang Y, Yew W-W. Mechanisms of drug resistance in Mycobacterium tuberculosis: update 2015. *Int J Tuberc Lung Dis* 2015; **19**: 1276–89.

9 Camus J-C, Pryor MJ, Médigue C, Cole ST. Re-annotation of the genome sequence of Mycobacterium tuberculosis H37Rv. *Microbiology (Reading, Engl)* 2002; **148**: 2967–73.

10 Miotto P, Tessema B, Tagliani E, *et al.* A standardised method for interpreting the association between mutations and phenotypic drug resistance in Mycobacterium tuberculosis. *Eur Respir J* 2017; **50**. DOI:10.1183/13993003.01354-2017.

11 Salamon H, Yamaguchi KD, Cirillo DM, *et al.* Integration of published information into a resistance-associated mutation database for Mycobacterium tuberculosis. *J Infect Dis* 2015; **211 Suppl 2**: S50-57.

12 Coll F, McNerney R, Guerra-Assunção JA, *et al.* A robust SNP barcode for typing Mycobacterium tuberculosis complex strains. *Nat Commun* 2014; **5**: 4812.

13 Avalos E, Catanzaro D, Catanzaro A, *et al.* Frequency and geographic distribution of gyrA and gyrB mutations associated with fluoroquinolone resistance in clinical Mycobacterium tuberculosis isolates: a systematic review. *PLoS ONE* 2015; **10**: e0120470.

14 Sandgren A, Strong M, Muthukrishnan P, Weiner BK, Church GM, Murray MB. Tuberculosis drug resistance mutation database. *PLoS Med* 2009; **6**: e2.

15 Broad Institute - Tuberculosis Drug Resistance Mutation Database. http://www.broadinstitute.org/annotation/genome/mtb_drug_resistance.1/DirectedSequencingHome.html (accessed December 29, 2017). .

Supplement Tables

Supplement Table 1. Laboratory methods for isolation of *M. tuberculosis*, phenotypic susceptibility testing and sequencing by country.

Country	Number of isolates	Isolation of M. tuberculosis and phenotypic susceptibility testing		Sequencing method
		Löwenstein-Jensen	MGIT 960	
Azerbaijan	751	*M.tb* isolation, DST to: RIF, INH	DST to: OFX, MFX, PZA, AMK, CAP	WGS using Illumina
Bangladesh	949	*M.tb* isolation, DST to: RIF, INH	DST to: OFX, MFX, PZA, KAN	WGS using Illumina
Belarus	197		*M.tb* isolation, DST to: RIF, INH, OFX, MFX, PZA, KAN, AMK, CAP	WGS using Ion PGM
Pakistan	1,461	*M.tb* isolation, DST to: RIF, INH	DST to: OFX, MFX, PZA, KAN, CAP	Sanger or WGS using Illumina *
Philippines	1,017	*M.tb* isolation, DST to: RIF, INH		Sanger
South Africa	1,578		*M.tb* isolation, DST to: RIF, INH, OFX, MFX, PZA, KAN, AMK, CAP	WGS using Illumina
Ukraine	1,141	*M.tb* isolation, DST to: RIF, INH, OFX, KAN, AMK, CAP	DST to: MFX	WGS using Illumina

LJ: Lowenstein-Jensen; DST: drug susceptibility testing; RIF: rifampicin; INH: isoniazid; OFX: ofloxacin; MFX: moxifloxacin; PZA: pyrazinamide; KAN: kanamycin; AMK: amikacin; CAP: capreomycin; WGS: whole genome sequencing.

* of the 1,461 isolates from Pakistan, 1,254 underwent Sanger sequencing and the remaining 207 underwent WGS using Illumina

Supplement Table 2. List of primers used for amplification of the genomic regions sequenced by Sanger technique.

Drug resistance	Locus tag	Gene ID	Forward primer sequence (5'-3')	Reverse primer sequence (5'-3')	Length (bp)
Pyrazinamide	Rv2043c + promoter	*pncA*	GCTGGTCATGTTCGCGATCG	CAGGAGCTGCAAACCAACTCG	664
Fluoroquinolones	Rv0006	*gyrA* QRDR	GATGACAGACACGACGTTGC	GGGCTTCGGTGTACCTCAT	398
Fluoroquinolones	Rv0005	*gyrB* QRDR	GTCGTTGTGAACAAGGCTGTG	GTGGAAATATGTTGGCCGTC	413
Rifampicin	Rv0667	*rpoB* hotspot	GGGAGCGGATGACCACCCA	GCGGTACGGCGTTTCGATGAAC	350
Rifampicin	Rv0667	*rpoB* N-terminal	CGACGAGTGCAAAGACAAGGACA	GACGGTGTCGCGCTTGTCGAC	310
Isoniazid	Rv1908c	*katG* codon 315	CGGCGCATGGCCATGAACGACGTC	CCGGCACCGGCGCCGTCCTTG	329
Isoniazid	Rv1484	*inhA*	CTATATCTCCGGTGCGGTCAT	TCGGGCCCTTCCGGTC	941
Isoniazid	Rv1483 promoter	*fabG* promoter	CGAAGTGTGCTGAGTCACACCG	CGATCCCCCGGTTTCCTCCG	219

QRDR: quinolone resistance-determining region.

Supplement Table 3. Number of clinical *M. tuberculosis* isolates included in the study and the pooled sensitivity values stratified by rifampicin resistance status for each respective locus or loci.

Drug	Locus	Country	RIF-susceptible cases		RIF-resistant cases		All cases	
			no. of isolates	sensitivity % (95%CI)	no. of isolates	sensitivity % (95%CI)	no. of isolates	sensitivity % (95%CI)
rifampicin	*rpoB*	Azerbaijan	-	-	-	-	745	95 (90-98)
		Bangladesh	-	-	-	-	941	86 (76-93)
		Belarus	-	-	-	-	191	93 (86-97)
		Pakistan	-	-	-	-	1,455	85 (76-91)
		Philippines	-	-	-	-	1,003	89 (81-95)
		South Africa	-	-	-	-	1,535	87 (79-94)
		Ukraine	-	-	-	-	1,140	94 (91-96)
isoniazid	*katG* & *inhA*	Azerbaijan	616	81 (72-88)	129	85 (78-90)	745	83 (78-88)
		Bangladesh	871	94 (82-99)	70	84 (73-92)	941	88 (81-93)
		Belarus	97	90 (70-99)	94	99 (94-100)	191	97 (93-99)
		Pakistan	1,353	65 (56-73)	99	75 (65-83)	1,452	69 (62-75)
		Philippines	937	80 (72-87)	76	90 (80-96)	1,013	84 (78-89)
		South Africa	1,464	48 (37-59)	76	84 (72-93)	1,540	62 (54-70)
		Ukraine	781	92 (86-96)	355	97 (95-98)	1,136	96 (93-97)
ofloxacin	*gyrA* & *gyrB*	Azerbaijan	615	67 (9-99)	130	85 (69-95)	745	84 (68-94)
		Bangladesh	860	95 (84-99)	68	100 (71-100)	928	96 (87-100)
		Belarus	97	0 (0-85)	93	93 (76-99)	190	87 (69-96)
		Pakistan	511	73 (65-81)	96	86 (65-97)	607	75 (68-82)
		South Africa	1,426	50 (1-99)	77	83 (52-98)	1,503	79 (49-95)
		Ukraine	782	83 (36-100)	355	90 (80-96)	1,137	89 (80-95)
moxifloxacin	*gyrA* & *gyrB*	Azerbaijan	615	100 (15-100)	130	88 (72-97)	745	89 (73-97)
		Bangladesh	860	98 (87-100)	68	100 (71-100)	928	98 (90-100)
		Belarus	97	0 (0-85)	93	89 (71-96)	190	83 (64-94)
		Pakistan	511	80 (72-87)	96	95 (75-100)	607	82 (75-89)
		South Africa	1,416	50 (1-99)	77	91 (59-100)	1,493	85 (55-98)
		Ukraine	555	100 (48-100)	275	92 (82-98)	830	93 (83-98)
pyrazinamide	*pncA*	Azerbaijan	616	58 (28-85)	129	54 (43-65)	745	55 (44-65)
		Bangladesh	49	31 (11-59)	24	40 (19-64)	73	36 (21-54)
		Belarus	99	75 (19-99)	98	83 (72-90)	197	82 (72-90)
		Pakistan	98	25 (3-65)	46	35 (20-54)	144	33 (20-50)
		South Africa	1,477	24 (11-41)	76	64 (45-80)	1,553	43 (31-56)
		Ukraine	-	-	281	48 (40-55)	-	-
pyrazinamide*	*pncA*	Azerbaijan	616	70 (35-93)	129	54 (43-65)	745	56 (45-66)
		Bangladesh	49	33 (12-62)	24	38 (18-62)	73	36 (21-54)
		Belarus	99	75 (19-99)	98	81 (71-89)	197	81 (71-89)
		Pakistan	98	29 (4-71)	46	36 (20-55)	144	35 (21-52)
		South Africa	1,477	57 (29-82)	76	60 (42-76)	1,553	59 (44-73)
		Ukraine	-	-	281	48 (40-55)	-	-
kanamycin	*rrs* & *eis*	Belarus	-	-	97	89 (73-97)	-	-
		Pakistan	-	-	74	100 (15-100)	-	-
		South Africa	-	-	83	100 (15-100)	-	-
		Ukraine	-	-	369	67 (58-75)	-	-
amikacin	*rrs*	Azerbaijan	-	-	140	86 (71-95)	-	-
		Belarus	-	-	97	92 (74-99)	-	-
		South Africa	-	-	84	67 (9-99)	-	-
		Ukraine	-	-	369	95 (86-99)	-	-
capreomycin	*rrs*	Azerbaijan	-	-	141	80 (65-91)	-	-
		Belarus	-	-	97	96 (76-100)	-	-
		Pakistan	-	-	73	67 (9-99)	-	-
		South Africa	-	-	84	33 (4-78)	-	-
		Ukraine	-	-	369	90 (80-96)	-	-

*adjusted with Wayne test results; CI - confidence interval

Supplement Table 4. Adjusted prevalence of resistance as measured using genetic sequencing and true prevalence of resistance determined using phenotypic testing.

Drug	Locus	Country	adjusted prevalence of resistance						true prevalence of resistance					
			RIF-susceptible cases		RIF-resistant cases		All cases		RIF-susceptible cases		RIF-resistant cases		All cases	
			%	(95%CI)	%	(95%CI)	%	(95%CI)	%	(95%CI)	%	(95%CI)	%	(95%CI)
rifampicin	*rpoB*	Azerbaijan	-	-	-	-	20.4	(17.3 - 23.7)	-	-	-	-	19.3	(16.6 - 22.4)
		Bangladesh	-	-	-	-	7.3	(5.6 - 9.1)	-	-	-	-	7.5	(5.9 - 9.4)
		Belarus	-	-	-	-	53.8	(45.8 - 61.9)	-	-	-	-	52.4	(45 - 59.6)
		Pakistan	-	-	-	-	6.5	(5.2 - 7.9)	-	-	-	-	6.9	(5.6 - 8.3)
		Philippines	-	-	-	-	8.3	(6.6 - 10.3)	-	-	-	-	8.4	(6.7 - 10.3)
		South Africa	-	-	-	-	5.5	(4.4 - 6.8)	-	-	-	-	5.7	(4.6 - 6.9)
		Ukraine	-	-	-	-	34.0	(30.8 - 37.3)	-	-	-	-	32.6	(29.9 - 35.4)
isoniazid	*katG* & *inhA*	Azerbaijan	17.8	(13.9 - 22.7)	91.2	(82.4 - 99)	32.1	(27.3 - 38.2)	17.5	(14.5 - 20.7)	97.2	(93 - 99.2)	32.9	(29.5 - 36.4)
		Bangladesh	6.5	(4.6 - 8.8)	89.5	(77.8 - 98.9)	12.8	(10.2 - 15.8)	5.4	(4.0 - 7.1)	97.2	(90.2 - 99.7)	12.3	(10.3 - 14.6)
		Belarus	26.4	(16.4 - 38.6)	98.6	(95.2 - 100)	70.2	(59.8 - 83.6)	22.6	(14.6 - 32.4)	99.0	(94.4 - 100)	61.8	(54.5 - 68.7)
		Pakistan	7.6	(5.8 - 9.8)	74.9	(63.4 - 87.1)	12.1	(10.0 - 14.7)	9.3	(7.8 - 10.9)	90.1	(82.5 - 95.1)	14.9	(13.1 - 16.8)
		Philippines	13.2	(10.3 - 16.8)	83.8	(71.7 - 95.8)	18.4	(15.3 - 22.3)	13.0	(10.9 - 15.3)	84.1	(74.4 - 91.3)	18.8	(16.4 - 21.3)
		South Africa	3.5	(2.5 - 4.9)	61.6	(49.4 - 74.3)	6.7	(5.3 - 8.5)	5.8	(4.6 - 7.1)	65.5	(54.6 - 75.4)	9.2	(7.8 - 10.7)
		Ukraine	20.0	(16.1 - 25.1)	98.6	(95.8 - 100)	48.4	(42.8 - 56.1)	17.4	(14.7 - 20.2)	97.3	(95.1 - 98.7)	43.4	(40.5 - 46.3)
ofloxacin	*gyrA* & *gyrB*	Azerbaijan	0.7	(0.1 - 1.7)	23.1	(16.2 - 31.1)	5.1	(3.5 - 7.0)	0.5	(0.1 - 1.5)	23.4	(16.8 - 31.2)	5.0	(3.5 - 6.8)
		Bangladesh	6.6	(4.3 - 10.1)	19.2	(10.4 - 30.0)	6.6	(4.9 - 8.6)	4.9	(3.5 - 6.6)	15.9	(8.2 - 26.7)	5.7	(4.3 - 7.4)
		Belarus	1.4	(0 - 5.3)	30.9	(21.4 - 41.6)	16.6	(11.2 - 23.0)	2.2	(0.3 - 7.6)	28.9	(20.1 - 39)	15.8	(10.9 - 21.8)
		Pakistan	24.8	(18.0 - 35.8)	22.9	(14.7 - 32.6)	21.5	(17.7 - 25.8)	24.3	(20.6 - 28.3)	22.7	(14.8 - 32.3)	24.1	(20.7 - 27.7)
		South Africa	0.3	(0 - 1.0)	13.9	(7.2 - 22.6)	0.9	(0.5 - 1.5)	0.2	(0 - 0.9)	13.6	(7.2 - 22.6)	0.9	(0.5 - 1.6)
		Ukraine	1.1	(0.4 - 2.2)	18.6	(14.4 - 23.2)	6.8	(5.3 - 8.6)	0.8	(0.3 - 1.7)	18.1	(14.3 - 22.4)	6.4	(5.1 - 8.0)
moxifloxacin	*gyrA* & *gyrB*	Azerbaijan	0.6	(0.1 - 1.6)	22.5	(15.7 - 30.2)	4.9	(3.4 - 6.7)	0.3	(0 - 1.2)	22.8	(16.2 - 30.5)	4.7	(3.3 - 6.5)
		Bangladesh	6.1	(4.0 - 9.5)	18.7	(10.1 - 29.2)	6.4	(4.8 - 8.2)	4.8	(3.4 - 6.4)	15.9	(8.2 - 26.7)	5.6	(4.2 - 7.3)
		Belarus	1.4	(0 - 5.1)	27.8	(19.0 - 37.7)	14.9	(9.8 - 20.9)	2.2	(0.3 - 7.6)	27.8	(19.2 - 37.9)	15.3	(10.5 - 21.2)
		Pakistan	22.7	(16.6 - 33.4)	22.3	(14.3 - 31.7)	20.5	(16.9 - 24.4)	21.8	(18.3 - 25.6)	20.6	(13.1 - 30.0)	21.6	(18.4 - 25.1)
		South Africa	0.3	(0 - 0.9)	13.6	(7.1 - 21.9)	0.9	(0.5 - 1.5)	0.3	(0 - 0.9)	12.6	(6.5 - 21.5)	0.9	(0.5 - 1.5)
		Ukraine	1.4	(0.5 - 3.0)	19.0	(14.4 - 24.1)	7.6	(5.7 - 9.7)	0.9	(0.3 - 2.1)	18.4	(14.1 - 23.4)	7.0	(5.3 - 8.9)
pyrazinamide	*pncA*	Azerbaijan	2.8	(1.1 - 5.5)	59.9	(41.4 - 84.6)	13.7	(8.9 - 21.2)	1.7	(0.8 - 3.0)	57.6	(49.1 - 65.8)	12.5	(10.2 - 15.1)
		Bangladesh	24.8	(8.8 - 50.5)	63.3	(31.8 - 95.4)	36.1	(18.9 - 61.9)	30.6	(18.3 - 45.4)	87.5	(67.6 - 97.3)	49.3	(37.4 - 61.3)
		Belarus	8.7	(2.2 - 19.9)	93.8	(81.4 - 99.8)	66.8	(46.5 - 93.3)	4.2	(1.2 - 10.4)	79.4	(70.3 - 86.8)	43.1	(36.1 - 50.4)
		Pakistan	6.3	(1.3 - 15.9)	51.5	(27.9 - 83.0)	19.9	(10.3 - 34.1)	7.1	(2.9 - 14.2)	71.7	(56.5 - 84.0)	27.8	(20.6 - 35.9)
		South Africa	1.3	(0.5 - 2.5)	47.7	(29.3 - 72.4)	3.7	(2.2 - 6.1)	1.0	(0.5 - 1.6)	40.7	(30.2 - 51.8)	3.2	(2.3 - 4.2)
		Ukraine	-	-	57.2	(41.6 - 79.1)	-	-	-	-	62.6	(56.7 - 68.3)	-	-
kanamycin	*rrs* & *eis*	Belarus	-	-	42.1	(28.7 - 60.6)	-	-	-	-	36.1	(26.6 - 46.5)	-	-
		Pakistan	-	-	5.2	(1.1 - 12.5)	-	-	-	-	2.7	(0.3 - 9.4)	-	-
		South Africa	-	-	9.6	(2.0 - 22.8)	-	-	-	-	5.1	(0.6 - 17.3)	-	-
		Ukraine	-	-	29.5	(22.3 - 40.1)	-	-	-	-	33.6	(28.8 - 38.7)	-	-
amikacin	*rrs*	Azerbaijan	-	-	25.9	(18.5 - 34.4)	-	-	-	-	26.4	(19.3 - 34.5)	-	-
		Belarus	-	-	27.0	(18.1 - 37)	-	-	-	-	25.8	(17.4 - 35.7)	-	-
		South Africa	-	-	3.9	(0.8 - 9.2)	-	-	-	-	3.6	(0.7 - 10.1)	-	-
		Ukraine	-	-	17.1	(13.1 - 21.6)	-	-	-	-	16.0	(12.4 - 20.1)	-	-
capreomycin	*rrs*	Azerbaijan	-	-	30.5	(20.4 - 45.2)	-	-	-	-	29.1	(21.7 - 37.3)	-	-
		Belarus	-	-	31.0	(19.5 - 47)	-	-	-	-	24.7	(16.5 - 34.5)	-	-
		Pakistan	-	-	5.1	(1.0 - 12.4)	-	-	-	-	4.1	(0.9 - 11.5)	-	-
		South Africa	-	-	4.5	(0.9 - 10.9)	-	-	-	-	7.1	(2.7 - 14.9)	-	-
		Ukraine	-	-	19.7	(14 - 28.6)	-	-	-	-	16.8	(13.1 - 21)	-	-

CI - confidence interval

Supplement Table 5. Mutations in *rpoB* gene associated with phenotypic resistance to rifampicin.

rpoB **mutation (codon position)**	**No. of isolates with adjusted resistant phenotype**	**No. of isolates with adjusted susceptible phenotype**	**No. of isolates with resistant phenotype**	**No. of isolates with susceptible phenotype**	**Medium used in discordant cases**	**Grading system**	**% on total RIF-R (total=958)**
Val170Phe*	5	0	5	0		Indeterminate	0.5
Glu423Ala + Ser450Trp	1	0	1	0		High	0.1
Del 425	0	1	0	1	1 LJ	Indeterminate	0.0
Leu430Pro	30	0	10	20	8 LJ, 12 MGIT	Minimal	3.1
Leu430Pro + Asp435Gly	1	0	1	0		High	0.1
Leu430Pro + Asp435Tyr	1	0	1	0		Moderate	0.1
Leu430Pro + His445Asn	1	0	1	0		High	0.1
Leu430Pro + His445Gln	3	0	3	0		Minimal	0.3
Leu430Pro + Met434Ile	1	0	1	0		Minimal	0.1
Leu430Pro + Met434Val	1	0	1	0		Minimal	0.1
Ins 430	1	0	1	0		Indeterminate	0.1
Ins 430 + Asp435Gly	1	0	1	0		High	0.1
Ins 431	1	1	1	1	1 LJ	Indeterminate	0.1
Gln432Glu + Glu481Ala	1	0	1	0		Indeterminate	0.1
Gln432Leu	1	0	1	0		High	0.1
Gln432Lys	3	0	2	1	1 MGIT	High	0.3
Gln432Pro	2	0	2	0		High	0.2
Del 432	2	0	2	0		Indeterminate	0.2
Ins 432	2	0	2	0		Indeterminate	0.2
Ins 433	2	0	2	0		Indeterminate	0.2
Met434Leu + His445Asn	1	0	0	1	1 LJ	Minimal	0.1
Met434Val + Asp435Gly	1	0	0	1	1 LJ	High	0.1
Asp435Ala + Leu452Pro	1	0	1	0		High	0.1
Asp435Glu + Ser441Leu	1	0	1	0		Moderate	0.1
Asp435Glu + His445Asn	1	0	1	0		Minimal	0.1
Asp435Gly	2	0	2	0		High	0.2
Asp435Gly + His445Asn	1	0	1	0		High	0.1
Asp435Gly + Leu452Pro	1	0	1	0		High	0.1
Asp435Phe	4	0	4	0		High	0.4
Asp435Tyr	24	0	12	12	4 LJ, 8 MGIT	Moderate	2.5
Asp435Val	35	0	30	5	5 LJ	High	3.7
Ser441Gln	1	0	1	0		High	0.1
Ser441Leu	2	0	1	1	1 LJ	Moderate	0.2
His445Arg	15	0	14	1	1 LJ	High	1.6
His445Asn	10	0	4	6	5 LJ, 1 MGIT	Minimal	1.0
His445Asn + Ile491Met	1	0	1	0		Minimal	0.1
His445Asp	50	0	46	4	2 LJ, 2 MGIT	High	5.2
His445Cys	1	0	1	0		High	0.1
His445Leu	22	0	18	4	3 LJ, 1 MGIT	High	2.3
His445Phe	1	0	1	0		Indeterminate	0.1
His445Ser	0	2	0	2	2 LJ	Indeterminate	0.0
His445Ser + Lys446Gln	1	0	1	0		Indeterminate	0.1
His445Thr	1	0	1	0		Indeterminate	0.1
His445Tyr	34	0	31	3	3 LJ	High	3.5
Arg448Lys	1	0	1	0		Indeterminate	0.1
Del 449	1	0	1	0		Indeterminate	0.1
Ser450Cys	1	1	1	1	1 MGIT	Indeterminate	0.1
Ser450Leu	541	0	530	11	7 LJ, 4 MGIT	High	56.5
Ser450Leu + Ile491Val	3	0	3	0		High	0.3
Ser450Leu + Leu452Pro	1	0	1	0		High	0.1
Ser450Phe	38	0	38	0		High	4.0
Ser450Trp	9	0	9	0		High	0.9
Leu452Pro	27	0	12	15	8 LJ, 7 MGIT	Moderate	2.8
Ile491Phe*	5	0	3	2	2 MGIT	Minimal	0.5
Ins 849*	0	1	0	1	1 LJ	Indeterminate	0.0
WT**	60	6046				-	6.3
**TOTAL**	**958**	**6052**					**100.0**

* mutations outside Rifampicin Resistance-Determining Region (RRDR); Ins: insertion; Del: deletion

**WT: includes wild-type, silent mutations and phylogenetically informative variants

Supplement Table 6. Mutations in *katG* and *inhA* genes and *fabG* promoter associated with phenotypic resistance to isoniazid.

katG mutation (codon position)	*inhA* mutation (codon position)	No. of isolates with adjusted resistant phenotype	No. of isolates with adjusted susceptible phenotype	No. of isolates with resistant phenotype	No. of isolates with susceptible phenotype	Medium used in discordant cases	Grading system	% on total INH-R (total=1519)
Ins 6 + Del 10	wt	1	0	1	0		Indeterminate	0.1
Ins 41	wt	1	0	1	0		Indeterminate	0.1
Ins 92	wt	1	0	1	0		Indeterminate	0.1
Asp94Ala	wt	1	0	1	0		Indeterminate	0.1
Asp94Tyr + Ala621Asp	wt	1	0	1	0		Indeterminate	0.1
His108Arg	wt	1	0	1	0		Indeterminate	0.1
Ala109Val	wt	2	0	2	0		Indeterminate	0.1
Gly125Asp	wt	1	0	1	0		Indeterminate	0.1
Met126Ile + Trp191Gly	wt	1	0	1	0		Indeterminate	0.1
Del 131	wt	1	0	1	0		Indeterminate	0.1
Asn138Asp	wt	1	0	1	0		Indeterminate	0.1
Ser140Asn + Asp142Gly	wt	1	0	1	0		Indeterminate	0.1
Leu141Phe	wt	2	0	2	0		Indeterminate	0.1
Asp142Gly	wt	1	1	1	1	1 MGIT	Indeterminate	0.1
Trp149Arg	wt	1	0	1	0		Indeterminate	0.1
Ins 153 + Ser315Arg	wt	1	0	1	0		Indeterminate	0.1
Asp163Asn	wt	1	0	1	0		Indeterminate	0.1
Gly169Val	wt	1	0	1	0		Indeterminate	0.1
Met176Ile	wt	1	0	1	0		Indeterminate	0.1
Val196Ala + Leu514Pro	wt	1	0	1	0		Indeterminate	0.1
Trp198Stop	wt	1	0	1	0		Indeterminate	0.1
Del 199	wt	1	0	1	0		Indeterminate	0.1
Thr251Pro	wt	1	0	1	0		Indeterminate	0.1
Thr275Pro	wt	1	0	1	0		Indeterminate	0.1
Gly279Asp	wt	2	0	2	0		Indeterminate	0.1
Pro280Ser	wt	0	8	0	8		Indeterminate	0.0
Leu298Ser	wt	1	0	1	0		Indeterminate	0.1
Gly299Ser	wt	2	0	2	0		Indeterminate	0.1
Trp300Gly	wt	1	0	1	0		Indeterminate	0.1
Ser315Arg	wt	2	0	2	0		Indeterminate	0.1
Ser315Asn	wt	7	0	7	0		High	0.5
Ser315Asp	wt	2	0	2	0		Indeterminate	0.1
Ser315Gly	wt	1	1	1	1	1 LJ	Indeterminate	0.1
Ser315Ile	wt	1	0	1	0		Indeterminate	0.1
Ser315Thr	wt	885	0	855	30	28 LJ, 2 MGIT	High	58.3
Glu318Ala	wt	1	0	1	0		Indeterminate	0.1
Trp321Stop	wt	1	0	1	0		Indeterminate	0.1
Thr324Pro	wt	1	0	1	0		Indeterminate	0.1
Ins 335	wt	1	0	1	0		Indeterminate	0.1
Thr344Pro	wt	1	0	1	0		Indeterminate	0.1
Thr376Gln + Met377Val	wt	1	0	1	0		Indeterminate	0.1
Thr380Ile	wt	1	0	1	0		Indeterminate	0.1
Ins380	wt	1	0	1	0		Indeterminate	0.1
Arg418Stop	wt	2	0	2	0		Indeterminate	0.1
Asp419Tyr	wt	1	0	1	0		Indeterminate	0.1
Del 428	wt	1	0	1	0		Indeterminate	0.1
Del 458	wt	1	0	1	0		Indeterminate	0.1
Gln461Pro	wt	1	0	1	0		Indeterminate	0.1
Del 480	wt	1	0	1	0		Indeterminate	0.1
Gly494Ala	wt	1	0	1	0		Indeterminate	0.1
Arg496Cys + Asn508Asp	wt	1	0	1	0		Indeterminate	0.1
Ins 669	wt	1	0	1	0		Indeterminate	0.1
Ser671Stop	wt	1	0	1	0		Indeterminate	0.1
Leu704Ser	wt	1	0	1	0		Indeterminate	0.1
wt	Del -34	1	0	1	0		Indeterminate	0.1
wt	g-17t	2	3	2	3	3 LJ	Indeterminate	0.1
wt	c-15t	171	0	148	23	19 LJ, 4 MGIT	Moderate	11.3
wt	c-15t + g-17t	1	0	1	0		Moderate	0.1
wt	c-15t + Ile21Val	1	0	1	0		Moderate	0.1
wt	c-15t + Ser94Ala	2	0	2	0		Moderate	0.1
wt	c-15t + Ile194Thr	1	0	1	0		Moderate	0.1
wt	t-8a	3	6	3	6	5 LJ, 1 MGIT	Indeterminate	0.2
wt	t-8a + Ile194Thr	1	0	1	0		Indeterminate	0.1
wt	t-8c	4	9	4	9	6 LJ, 3 MGIT	Indeterminate	0.3
wt	t-8g	1	0	1	0		Indeterminate	0.1
wt	Ile21Thr	2	0	2	0		Indeterminate	0.1
wt	Ile21Val	1	2	1	2	2 LJ	Indeterminate	0.1
wt	Ser94Ala	2	1	2	1	1 LJ	Indeterminate	0.1
wt	Ile194Thr	1	0	1	0		Indeterminate	0.1
Pro280Ser	c-15t	1	0	1	0		Moderate	0.1
Ser315Gly	c-15t	1	0	1	0		Moderate	0.1
Ser315Thr	g-17t	1	0	1	0		High	0.1
Ser315Thr	c-15t	182	0	179	3	3 LJ	High	12.0
Ser315Thr	t-8a	9	0	8	1	1 MGIT	High	0.6
Ser315Thr	t-8c	23	0	23	0		High	1.5
Ser315Thr	t-8c + c-15t	1	0	1	0		High	0.1
Ser315Thr	Ser94Ala	1	0	1	0		High	0.1
Tyr413Cys	c-15t	1	0	1	0		Moderate	0.1
WT**		155	5468				-	10.2
**TOTAL**		**1519**	**5499**					**100.0**

Ins: insertion; Del: deletion

**WT: includes wild-type, silent mutations and phylogenetically informative variants

Supplement Table 7. Mutations in *pncA* gene associated with phenotypic resistance to pyrazinamide.

pncA mutation (codon position)	No. of isolates with adjusted resistant phenotype	No. of isolates with adjusted susceptible phenotype	No. of isolates with resistant phenotype	No. of isolates with susceptible phenotype	No. of isolates with Pzase negative	No. of isolates with Pzase positive	Grading system	% on total PZA-R (total=500)	Indetermined mutations - role according to literature
a-15c	0	1	0	1	1	0	Indeterminate	0.0	unknown (unclear role)
a-15c + Leu4Ser	2	0	2	0	0	0	Indeterminate	0.4	resistant
a-15c + Ser67Pro	1	0	1	0	0	0	High	0.2	
t-12g	1	0	1	0	0	0	Indeterminate	0.2	likely resistant
a-11c	3	0	3	0	0	0	Indeterminate	0.6	likely resistant
a-11g	29	0	29	0	0	0	High	5.8	
a-11g + Ile6Leu	1	0	1	0	0	0	High	0.2	
Del -7*	1	0	1	0	1	0	Indeterminate	0.2	likely resistant
Del -5	1	0	1	0	0	0	High	0.2	
Met1Thr	3	0	3	0	0	0	Indeterminate	0.6	resistant
Ala3Pro + Leu182Ser	1	0	1	0	0	0	Indeterminate	0.2	resistant
Del 3	1	0	1	0	0	0	High	0.2	
Leu4Ser	2	0	2	0	0	0	Indeterminate	0.4	resistant
Leu4Stop	1	0	1	0	0	0	High	0.2	
Leu4Trp	2	0	2	0	0	0	Indeterminate	0.4	resistant
Ile5Thr	1	0	1	0	0	0	Indeterminate	0.2	resistant
Val7Ala	2	0	2	0	0	0	Indeterminate	0.4	likely resistant
Val7Gly	9	0	9	0	0	0	High	1.8	
Del 7	1	0	1	0	0	0	High	0.2	
Asp8Ala	1	0	1	0	0	0	Indeterminate	0.2	resistant
Val9Gly	2	0	2	0	2	0	Indeterminate	0.4	resistant
Gln10Arg	3	0	3	0	0	0	Indeterminate	0.6	resistant
Gln10His	1	0	1	0	0	0	Indeterminate	0.2	likely resistant
Gln10His + Asn11His	1	0	1	0	0	0	Indeterminate	0.2	likely resistant
Gln10Pro	3	0	3	0	0	0	High	0.6	
Gln10Stop	2	0	1	1	0	0	High	0.4	
Asp12Ala	3	0	2	1	0	1	High	0.6	
Asp12Asn	1	0	1	0	0	0	High	0.2	
Phe13Leu	1	0	1	0	0	0	Indeterminate	0.2	resistant
Cys14Arg	6	0	6	0	6	0	High	1.2	
Cys14Gly	0	1	0	1	0	0	Indeterminate	0.0	likely susceptible
Del 16	2	0	2	0	0	0	High	0.4	
Ins 16	1	0	1	0	0	0	High	0.2	
Gly17Val	1	0	1	0	0	0	Indeterminate	0.2	unknown (unclear role)
Ser18Stop	4	0	4	0	0	0	High	0.8	
Leu19Pro	4	0	4	0	0	0	Indeterminate	0.8	resistant
Leu27Pro	1	0	1	0	0	0	Indeterminate	0.2	resistant
Leu27Pro + Thr100Pro	1	0	1	0	0	0	Indeterminate	0.2	resistant
Ile31Ser	6	0	6	0	0	0	Indeterminate	1.2	resistant
Ins 31	1	0	1	0	0	0	High	0.2	
Tyr34Asp	3	0	3	0	2	0	High	0.6	
Tyr34Ser	2	0	2	0	0	0	Indeterminate	0.4	unknown (unclear role)
Glu37Stop	1	0	1	0	0	0	High	0.2	
Del 40	1	0	1	0	1	0	High	0.2	
Ins 39	1	0	1	0	0	0	High	0.2	
Del 40 + Thr119Stop	1	0	1	0	0	0	High	0.2	
Del 42	2	0	2	0	0	0	High	0.4	
Thr47Asn	2	0	2	0	0	0	Indeterminate	0.4	unknown (new mutation)
Thr47Pro	1	0	1	0	0	0	Indeterminate	0.2	resistant
Lys48Glu	2	0	2	0	0	0	Indeterminate	0.4	resistant
Lys48Glu + Asp136Gly	1	0	1	0	0	0	Indeterminate	0.2	resistant
Asp49Asn	1	0	1	0	0	0	Indeterminate	0.2	resistant
Asp49Glu	1	0	1	0	0	0	Indeterminate	0.2	resistant
Asp49Gly	28	0	26	2	1	1	High	5.6	
Del 50	1	0	1	0	0	0	High	0.2	
Ins 50	1	0	1	0	0	0	High	0.2	
His51Arg	2	0	2	0	0	0	Indeterminate	0.4	resistant
His51Asp	1	0	1	0	0	0	Indeterminate	0.2	likely resistant
His51Gln	4	0	3	1	1	0	High	0.8	
His51Pro	1	0	1	0	0	0	Indeterminate	0.2	resistant
Del 54	1	0	1	0	0	0	High	0.2	
Pro54Leu	2	0	2	0	0	0	Moderate	0.4	
His57Arg	1	0	0	1	0	0	High	0.2	
His57Asp	7	0	7	0	1	0	High	1.4	resistant
His57Pro	1	0	1	0	0	0	Indeterminate	0.2	resistant
His57Tyr	2	0	2	0	0	0	High	0.4	
Phe58Cys + Val128Gly	1	0	1	0	0	0	High	0.2	
Phe58Ser	1	0	1	0	1	0	Indeterminate	0.2	likely resistant
Ser59Pro	1	0	1	0	1	0	Indeterminate	0.2	resistant
Ins 62	1	0	0	1	1	0	High	0.2	
Pro62Leu	3	0	3	0	0	0	Indeterminate	0.6	resistant
Asp63Ala	1	0	1	0	0	0	Indeterminate	0.2	resistant
Asp63Gly	3	0	2	1	0	0	High	0.6	
Tyr64Stop	1	0	1	0	0	0	High	0.2	
Ins 65*	1	0	1	0	0	0	Indeterminate	0.2	likely resistant
Ser67Pro	2	0	2	0	0	0	High	0.4	
Trp68Arg	2	0	2	0	0	0	High	0.4	
Trp68Gly	9	0	9	0	1	0	Moderate	1.8	
Trp68Ser	1	0	1	0	0	0	Indeterminate	0.2	likely resistant
Pro69Leu	3	0	3	0	0	0	Indeterminate	0.6	likely resistant
His71Gln	1	0	1	0	1	0	Indeterminate	0.2	resistant
His71Tyr	9	0	8	1	5	0	High	1.8	
Cys72Arg	1	0	1	0	0	0	High	0.2	
Ins 73	1	0	1	0	0	0	High	0.2	
Thr76Ile	2	0	2	0	1	0	Indeterminate	0.4	resistant
Thr76Pro	6	0	6	0	1	0	High	1.2	
Del 77	1	0	0	1	1	0	High	0.2	
Gly78Cys	1	0	1	0	0	0	Indeterminate	0.2	unknown (unclear role)
Ala79Glu	1	0	1	0	0	0	Indeterminate	0.2	unknown (unclear role)
Phe81Val	1	0	1	0	0	0	Indeterminate	0.2	resistant
Ins 81	1	0	1	0	0	0	High	0.2	
His82Arg	5	0	5	0	0	0	High	1.0	
Ins 82	2	0	2	0	0	0	High	0.4	
Leu85Arg	1	0	1	0	0	0	High	0.2	
Leu85Pro	7	0	7	0	0	0	High	1.4	
Ins 93*	1	0	1	0	0	0	Indeterminate	0.2	likely resistant
Val93Ala	1	1	1	1	0	0	Indeterminate	0.2	unknown (unclear role)
Val93Gly	1	0	1	0	0	0	Indeterminate	0.2	unknown (new mutation)

pncA mutation (codon position)	No. of isolates with adjusted resistant phenotype	No. of isolates with adjusted susceptible phenotype	No. of isolates with resistant phenotype	No. of isolates with susceptible phenotype	No. of isolates with Pzase negative	No. of isolates with Pzase positive	Grading system	% on total PZA-R (total=500)	Indetermined mutations - role according to literature
Val93Met	1	0	1	0	0	0	Indeterminate	0.2	unknown (unclear role)
Phe94Leu	2	0	2	0	0	0	High	0.4	
Phe94Ser	1	0	1	0	0	0	High	0.2	
Tyr95Stop	1	0	1	0	0	0	High	0.2	
Del 96	2	0	2	0	0	0	High	0.4	
Ins 96	3	0	3	0	0	0	High	0.6	
Lys96Arg	3	1	3	1	1	0	Indeterminate	0.6	resistant
Lys96Gln	1	0	1	0	0	0	Indeterminate	0.2	resistant
Lys96Glu	0	1	0	1	0	0	Indeterminate	0.0	resistant
Lys96Thr	1	0	1	0	0	0	Indeterminate	0.2	resistant
Gly97Arg	1	0	1	0	1	0	High	0.2	
Gly97Asp	4	0	4	0	3	0	High	0.8	
Gly97Cys	2	0	2	0	2	0	Indeterminate	0.4	resistant
Gly97Ser	3	0	3	0	0	0	High	0.6	
Del 100	1	0	1	0	1	0	High	0.2	
Ala102Pro	1	0	1	0	0	0	Indeterminate	0.2	resistant
Ala102Thr	1	0	1	0	0	0	Indeterminate	0.2	resistant
Ala102Val	1	1	1	1	0	0	Indeterminate	0.2	likely susceptible
Tyr103Cys	2	0	2	0	0	0	Indeterminate	0.4	resistant
Tyr103His	1	0	1	0	0	0	High	0.2	
Tyr103Ser	1	0	1	0	0	0	Indeterminate	0.2	likely resistant
Ser104Arg	4	0	4	0	0	0	High	0.8	
Gly105Asp	7	0	7	0	0	0	Indeterminate	1.4	resistant
Gly105Val	1	0	1	0	0	0	Indeterminate	0.2	unknown (unclear role)
Ins 105	1	0	1	0	0	0	High	0.2	
Phe106Leu	1	0	1	0	0	0	Indeterminate	0.2	unknown (unclear role)
Del 106	4	0	4	0	0	0	High	0.8	
Gly108Arg	2	0	2	0	0	0	High	0.4	
Gly108Glu	1	0	1	0	0	0	Indeterminate	0.2	likely resistant
Del 114	2	0	2	0	0	0	High	0.4	
Ins 114	1	0	1	0	0	0	High	0.2	
Trp119Cys	2	0	2	0	0	0	Indeterminate	0.4	resistant
Trp119Gly	1	0	1	0	0	0	Indeterminate	0.2	resistant
Trp119Leu	1	0	1	0	0	0	Indeterminate	0.2	likely resistant
Leu120Arg	2	0	2	0	0	0	Indeterminate	0.4	likely resistant
Arg121Pro	1	0	1	0	0	0	Indeterminate	0.2	resistant
Val125Phe	2	0	2	0	0	0	Indeterminate	0.4	resistant
Del 127	1	0	1	0	0	0	High	0.2	
Val128Gly	1	0	1	0	0	0	High	0.2	
Asp129Asn	1	0	1	0	0	0	Indeterminate	0.2	unknown (unclear role)
Del 129*	4	0	4	0	0	0	Indeterminate	0.8	likely resistant
Ins 130	2	0	2	0	0	0	High	0.4	
Val131Phe	1	0	1	0	0	0	Indeterminate	0.2	unknown (unclear role)
Ins 131	6	0	6	0	0	0	High	1.2	
Gly132Ala	1	1	1	1	2	0	Indeterminate	0.2	resistant
Gly132Ser	5	0	5	0	2	0	Indeterminate	1.0	resistant
Ile133Ser	1	0	1	0	0	0	Indeterminate	0.2	likely resistant
Ile133Thr	4	0	4	0	0	0	Indeterminate	0.8	resistant
Del 133	1	0	1	0	0	0	High	0.2	
Ala134Val	7	0	7	0	0	0	Indeterminate	1.4	resistant
Thr135Pro	1	0	1	0	1	0	High	0.2	
Asp136Asn	1	0	1	0	0	0	Indeterminate	0.2	likely resistant
Asp136Glu	0	1	0	1	1	0	Indeterminate	0.0	unknown (unclear role)
Asp136Val	1	0	1	0	0	0	Indeterminate	0.2	unknown (new mutation)
Ins 136	1	0	1	0	0	0	High	0.2	
His137Asn	1	0	1	0	0	0	Indeterminate	0.2	unknown (unclear role)
Ins 137	1	0	1	0	0	0	High	0.2	
Cys138Arg	3	0	3	0	1	0	Indeterminate	0.6	resistant
Cys138Ser	1	0	1	0	0	0	Indeterminate	0.2	likely resistant
Ins 138	1	0	1	0	0	0	High	0.2	
Val139Ala	4	0	4	0	0	0	Minimal	0.8	
Val139Gly	1	0	1	0	1	0	High	0.2	
Arg140Pro	1	0	1	0	1	0	Indeterminate	0.2	unknown (unclear role)
Gln141Pro	6	0	6	0	0	0	High	1.2	
Gln141Stop	1	0	1	0	0	0	High	0.2	
Thr142Ala	5	0	3	2	0	0	High	1.0	
Thr142Met	2	0	2	0	0	0	High	0.4	
Ala146Thr	4	0	4	0	0	0	Indeterminate	0.8	likely resistant
Ins 150	2	0	1	1	0	0	High	0.4	
Leu151Ser	5	1	5	1	4	0	Indeterminate	1.0	resistant
Ins 153	3	0	3	0	3	0	High	0.6	
Arg154Gly	1	1	1	1	1	0	Indeterminate	0.2	likely resistant
Arg154Met	1	0	1	0	0	0	Indeterminate	0.2	unknown (unclear role)
Val155Ala	1	0	1	0	0	0	Indeterminate	0.2	likely resistant
Val155Gly	1	0	1	0	0	0	High	0.2	
Val155Met	4	0	4	0	0	0	Indeterminate	0.8	likely resistant
Ins 157	1	0	1	0	0	0	High	0.2	
Ins 159	1	0	1	0	0	0	High	0.2	
Leu159Arg	1	0	1	0	0	0	Indeterminate	0.2	resistant
Thr160Pro	6	0	6	0	0	0	Indeterminate	1.2	resistant
Gly162Asp	1	0	1	0	0	0	Indeterminate	0.2	resistant
Val163Gly	2	0	2	0	0	0	Indeterminate	0.4	unknown (unclear role)
Ala165Thr	0	1	0	1	1	0	Indeterminate	0.0	unknown (new mutation)
Del 166	1	0	1	0	0	0	High	0.2	
Thr168Pro	1	0	1	0	0	0	Indeterminate	0.2	resistant
Ala171Thr	1	0	1	0	0	0	Indeterminate	0.2	unknown (unclear role)
Leu172Pro	6	0	6	0	1	0	High	1.2	
Ins 173	1	0	1	0	1	0	High	0.2	
Met175Arg	1	0	1	0	0	0	Indeterminate	0.2	likely resistant
Met175Ile	0	2	0	2	2	0	Indeterminate	0.0	resistant
Met175Thr	2	0	2	0	0	0	Indeterminate	0.4	resistant
Met175Val	2	0	2	0	0	0	High	0.4	
Thr177Pro	3	0	3	0	0	0	Indeterminate	0.6	unknown (unclear role)
Val180Phe	1	0	1	0	0	0	Indeterminate	0.2	resistant
Stop187Arg	1	0	1	0	0	0	Indeterminate	0.2	resistant
WT**	63	2480						12.6	
**TOTAL**	**500**	**2493**						**100.0**	

* inframe insertion or deletion (not graded); Pzase: pyrazinamidase; Ins: insertion; Del: deletion.
**WT: includes wild-type, silent mutations and phylogenetically informative variants

Supplement Table 8. Mutations in *gyrA* and *gyrB* genes associated with phenotypic resistance to fluoroquinolones.

		ofloxacin							moxifloxacin						
***gyrA* mutation (codon position)**	***gyrB* mutation (codon position)**	**No. of isolates with adjusted resistant phenotype**	**No. of isolates with adjusted susceptible phenotype**	**No. of isolates with resistant phenotype**	**No. of isolates with susceptible phenotype**	**Medium used in discordant cases**	**Grading system**	**% on total OFX-R (total= 353)**	**No. of isolates with adjusted resistant phenotype**	**No. of isolates with adjusted susceptible phenotype**	**No. of isolates with resistant phenotype**	**No. of isolates with susceptible phenotype**	**Medium used in discordant cases**	**Grading system**	**% on total MFX-R (total=318)**
Gly88Ala	wt	1	0	1	0		High	0.3	1	0	1	0		Indeterminate	0.3
Gly88Asp	wt	1	0	1	0		Indeterminate	0.3	1	0	1	0		Indeterminate	0.3
Gly88Cys	wt	1	0	1	0		High	0.3	1	0	1	0		Indeterminate	0.3
Gly88Cys + Asp94Gly	wt	1	0	1	0		High	0.3	1	0	1	0		High	0.3
Asp89Asn	wt	1	1	1	1	1 MGIT	Indeterminate	0.3	0	2	0	2	2 MGIT	Indeterminate	0.0
Ala90Val	wt	66	0	62	4	4 MGIT	High	18.7	64	0	31	33	33 MGIT	High	20.1
Ala90Val + Asp94Ala	wt	2	0	2	0		High	0.6	2	0	2	0		High	0.6
Ala90Val + Asp94Asn	wt	2	0	2	0		High	0.6	2	0	2	0		High	0.6
Ala90Val + Asp94Gly	wt	9	0	9	0		High	2.5	9	0	9	0		High	2.8
Ala90Val + Asp94Ser	wt	1	0	1	0		High	0.3	1	0	1	0		High	0.3
Ala90Val + Asp94Tyr	wt	1	0	1	0		High	0.3	1	0	1	0		High	0.3
Ala90Val + Ser91Pro	wt	3	0	3	0		High	0.8	3	0	2	1	1 MGIT	High	0.9
Ser91Pro	wt	15	0	14	1	1 LJ	High	4.2	15	0	9	6	6 MGIT	High	4.7
Asp94Ala	wt	21	0	18	3	1 LJ, 2 MGIT	High	5.9	18	0	9	9	9 MGIT	High	5.7
Asp94Asn	wt	26	0	26	0		High	7.4	26	0	26	0		High	8.2
Asp94Gly	wt	128	0	125	3	1 LJ, 2 MGIT	High	36.3	122	0	114	8	8 MGIT	High	38.4
Asp94His	wt	2	0	2	0		High	0.6	2	0	2	0		Indeterminate	0.6
Asp94Tyr	wt	8	0	7	1	1 MGIT	High	2.3	8	0	7	1	1 MGIT	High	2.5
wt	Arg446Cys*	1	1	1	1	1 MGIT	Indeterminate	0.3	0	2	0	2	2 MGIT	Indeterminate	0.0
wt	Asp461Asn	3	0	3	0		Indeterminate	0.8	2	1	2	1	1 MGIT	Indeterminate	0.6
wt	Asp461His	1	0	1	0		Indeterminate	0.3	0	1	0	1	1 MGIT	Indeterminate	0.0
wt	Ala467Thr	1	0	1	0		Indeterminate	0.3	0	1	0	1	1 MGIT	Indeterminate	0.0
wt	Asn499Thr	0	1	0	1	1 MGIT	Indeterminate	0.0	0	1	0	1	1 MGIT	Indeterminate	0.0
wt	Thr500Asn	2	2	2	2	2 MGIT	Indeterminate	0.6	1	3	1	3	3 MGIT	Indeterminate	0.3
wt	Glu501Asp	2	2	2	2	1 LJ, 1 MGIT	Indeterminate	0.6	3	1	3	1	1 MGIT	Indeterminate	0.9
wt	Ala504Thr*	0	1	0	1	1 MGIT	Indeterminate	0.0	0	1	0	1	1 MGIT	Indeterminate	0.0
Ala90Val	Glu501Asp	1	0	1	0		High	0.3	1	0	1	0		High	0.3
Asp94Ala	Asp461Ala	1	0	1	0		High	0.3	1	0	1	0		High	0.3
Asp94Ala	Asn499Thr	1	0	1	0		High	0.3	1	0	0	1	1 MGIT	High	0.3
Asp94Asn	Glu501Asp	1	0	1	0		High	0.3	1	0	1	0		High	0.3
Asp94Tyr	Asp461Asn	1	0	1	0		High	0.3	1	0	1	0		High	0.3
Asp94Gly	Ala504Val*	1	0	1	0		High	0.3	1	0	1	0		High	0.3
Asp94Gly	Thr500Pro	1	0	1	0		High	0.3	1	0	1	0		High	0.3
WT**	WT**	47	4749					13.3	28	4462					8.8
	**TOTAL**	**353**	**4757**					**100.0**	**318**	**4475**					**100.0**

* mutations outside Quinolone Resistance-Determining Region (QRDR); OFX: ofloxacin; MFX: moxifloxacin.

**WT: includes wild-type, silent mutations and phylogenetically informative variants

Supplement Table 9. Mutations in *rrs* and *eis* genes associated with phenotypic resistance to injectable agents: kanamycin, amikacin, capreomycin.

kanamycin

rrs mutation	*eis* mutation	No. of isolates with adjusted resistant phenotype	No. of isolates with adjusted susceptible phenotype	No. of isolates with resistant phenotype	No. of isolates with susceptible phenotype	Medium used in discordant cases	Grading system	% on total KAN-R (total=191)
a1401g	wt	81	0	80	1	1 MGIT	High	42.4
c1402t	wt	0	0	0	0			0.0
wt	g-37t	18	0	18	0		Indeterminate	9.4
wt	c-14t	15	0	11	4	1 LJ, 3 MGIT	High	7.9
wt	c-12t	42	0	14	28	9 LJ, 19 MGIT	Indeterminate	22.0
wt	g-10a	20	0	9	11	8 LJ, 3 MGIT	High	10.5
wt	g-10c	1	0	1	0		Indeterminate	0.5
wt	c-8a	0	3	0	3	3 LJ	Indeterminate	0.0
wt	Del -8	5	0	5	0		Indeterminate	2.6
wt	Del -7	1	0	1	0		Indeterminate	0.5
a1401g	c-12t	1	0	1	0		High	0.5
a1401g	g-10c	1	0	1	0		High	0.5
WT**	WT**	6	468					3.1
		**191**	**471**					**100.0**

amikacin

rrs mutation	*eis* mutation	No. of isolates with adjusted resistant phenotype	No. of isolates with adjusted susceptible phenotype	No. of isolates with resistant phenotype	No. of isolates with susceptible phenotype	Medium used in discordant cases	Grading system	% on total AMK-R (total=124)
a1401g	wt	113	0	108	5	3 LJ, 2 MGIT	High	91.1
c1402t	wt	0	1	0	1	1 MGIT	Indeterminate	0.0
WT**	WT**	11	565					8.9
		**124**	**566**					**100.0**

capreomycin

rrs mutation	*eis* mutation	No. of isolates with adjusted resistant phenotype	No. of isolates with adjusted susceptible phenotype	No. of isolates with resistant phenotype	No. of isolates with susceptible phenotype	Medium used in discordant cases	Grading system	% on total CAP-R (total=136)
a1401g	wt	115	0	92	23	20 LJ, 3 MGIT	High	84.6
c1402t	wt	1	0	1	0		High	0.7
WT**	WT**	20	628					14.7
		**136**	**628**					**100.0**

Del: deletion; KAN: kanamycin; AMK: amikacin; CAP: capreomycin.
**WT: includes wild-type, silent mutations and phylogenetically informative variants
